# A New Species of *Aleiodes* Wesmael (Braconidae, Rogadinae) with Potential for Biological Control of *Spodoptera* spp. (Lepidoptera, Noctuidae), and Notes on the Definition of the *gastritor, circumscriptus,* and Related Species-Groups

**DOI:** 10.1007/s13744-023-01076-8

**Published:** 2023-09-19

**Authors:** Eduardo Mitio Shimbori, Tamara Akemi Takahashi, Isabela Midori Watanabe, Angélica Maria Penteado-Dias, Luís Amilton Foerster, Scott Richard Shaw, José Roberto Postali Parra

**Affiliations:** 1 Depto de Entomologia e Acarologia, Escola Superior de Agricultura “Luiz de Queiroz” (ESALQ), Univ de São Paulo (USP), Piracicaba, SP Brazil; 2 Colección Nacional de Insectos, Instituto de Biología, Univ Nacional Autónoma de México, Mexico City, Mexico; 3 Depto de Ecologia e Biologia Evolutiva, Univ Federal de São Carlos, São Carlos, SP Brazil; 4 Depto de Zoologia, Univ Federal do Paraná, Curitiba, PR Brazil; 5 Dept of Ecosystem Science and Management, Univ Wyoming, Laramie, WY USA

**Keywords:** *Spodoptera frugiperda*, *Spodoptera cosmioides*, *Spodoptera eridania*, Taxonomy, Maize, Soybean

## Abstract

A new species of *Aleiodes* Wesmael in the *gastritor* Thunberg species-group is described and illustrated. The new species, *Aleiodes ceres* Shimbori **sp.n.**, occurs in Southern Brazil and Argentina. It is a potential biological control agent of caterpillars in the genus *Spodoptera* Guenée, namely *S. cosmioides* (Walker), *S. eridania* (Stoll), and *S. frugiperda* (J.E. Smith), which are serious pests on major crops including maize and soybean. The position of the new species within *Aleiodes* is discussed in relation to existing proposals of subdivisions of the genus into species-groups. In agreement with recent publications, the *gastritor* species-group is treated as separate from the *circumscriptus* Nees*/bicolor* Spinola group, as evidence suggests the latter is absent in the New World. We also propose the provisional *shakirae* Shimbori & Shaw species-group, to accommodate a clade of Neotropical species with geometrid hosts and morphological features similar to the *gastritor* and *circumscriptus* groups. A key to Neotropical species in the *gastritor* group and similar species is provided, except for species recently named without a morphological description.

**Supplementary Information:**

The online version contains supplementary material available at 10.1007/s13744-023-01076-8.

## Introduction

The genus *Aleiodes* Wesmael is the most diverse lineage within Rogadinae (Hymenoptera, Braconidae), and has a worldwide distribution. All rogadines are koinobiont endoparasitoids of lepidopteran larvae and mummify their host caterpillar while pupating inside it (Shaw and Huddleston 1991; Shaw 2006; Zaldívar-Riverón et al. 2008). Virtually, all *Aleiodes* species are solitary on exposed feeders in several lepidopteran families, with basal groups mainly associated with Noctuidae (Zaldívar-Riverón et al. 2008; van Achterberg and Shaw 2016).

The most comprehensive studies subdividing *Aleiodes* into species-groups form the foundation for studies on the systematics of the genus (Shaw 1997; Fortier and Shaw 1999). Two decades after that proposition, some groups had their monophyly contested, mostly based on molecular phylogenies (Zaldívar-Riverón et al. 2008; van Achterberg et al. 2020), reinterpreted in various ways (Townsend and Shaw 2009; van Achterberg and Shaw 2016), and additional subgroups have been proposed (Fortier 2006; Areekul-Butcher and Quicke 2011; Areekul-Butcher et al. 2012; Shimbori et al. 2016). These rearrangements and new species-groups are based on taxonomic studies, mainly of faunas of previously underrepresented biogeographical regions, such as the Oriental and Neotropical regions, with or without the inclusion of molecular data.

Currently, there are several isolated groups recovered as monophyletic in at least one molecular phylogeny (Quicke et al. 2006; Areekul-Butcher et al. 2012; van Achterberg et al. 2020), but the majority of the species in *Aleiodes* (precisely those that need major taxonomic efforts, such as the *seriatus* Herrich-Schaffer group sensu Fortier and Shaw 1999) are still laying outside these groups, especially in tropical faunas (Areekul-Butcher et al. 2012; Shaw et al. 2020). In this context, the Palearctic fauna is much better resolved, with revisions clarifying the systematics of the genus (van Achterberg and Shaw 2016; van Achterberg et al. 2020). Unfortunately, the subdivision system developed for the Palearctic is not entirely appropriate for the New World, and even for that fauna, there are several species not included in any group (van Achterberg and Shaw 2016). Because there is no recently published phylogeny testing monophyly of all subgroups of *Aleiodes*, or with enough coverage to propose a new subdivision to the worldwide fauna, the subdivisions based on morphology are still useful as a working framework, especially for those species-groups with unresolved phylogenetic relationships.

In the Neotropical region, the diversity of *Aleiodes* has proven to be one of the highest worldwide (Townsend and Shaw 2009; Shimbori et al. 2015, 2016; Garro et al. 2017; Shaw et al. 2020), and the fauna is especially diverse in two species-groups: the *seriatus* and the *circumscriptus/gastritor* Townsend & Shaw groups. The first of these is presumed to be the most diverse group in the neotropics and has recently received a revision for one of the many morphologically cohesive subgroups, the *bakeri* Brues species-subgroup (Shaw et al. 2020). The second group is a combination of two groups proposed after a major study on Neotropical *Aleiodes* (Townsend and Shaw 2009)*.* In that study, the authors highlighted the lack of a clear line defining the *gastritor* Thunberg and *circumscriptus* Nees groups in neotropical species, even though this line is clear in the Holarctic fauna (Shaw et al. 1997a, b; Fortier and Shaw 1999). The species-group defined as *circumscriptus/gastritor* (Townsend and Shaw 2009) comprises many undescribed species and is one of the major taxonomic challenges in the genus. Its diversity could be explained by rapid recent radiation (van Achterberg et al. 2020), one of the reasons why this is the only species-group not yet revised for the Nearctic region.

The moth genus *Spodoptera* Guenée (Lepidoptera, Noctuidae) comprises 31 species, of which 15 are considered pests in the Western and Eastern hemispheres (Kergoat et al. 2021). In Brazil, there are at least eight species of *Spodoptera*, namely *S. albula* (Walker, 1857), *S. androgea* (Stoll, 1782), *S. cosmioides* (Walker, 1858), *S. dolichos* (Fabricius, 1794), *S. eridania* (Stoll, 1782), *S. evanida* Schaus, 1914, *S. frugiperda* (J.E. Smith, 1797), and *S. ornithogalli* (Guenée, 1852) (Pogue 2002), and some of them are considered main pests on cotton, maize, and soybean fields (Martinelli et al. 2006). Since the years 2013/2014 soybean crop season, with the approval of the commercial release of genetically modified soybean expressing Cry1Ac protein from *Bacillus thuringiensis* Berliner (Bt) (CTNBio 2010), there has been an increase in the occurrence of *S. cosmioides*, *S. eridania*, and *S. frugiperda*. These outbreaks of *Spodoptera* species can be explained by the low susceptibility of these insects to genetically modified soybean expressing Cry1Ac protein from *Bt* (Bernardi et al. 2014). In maize, *S. frugiperda* is the most destructive and important pest in Brazil (Cruz et al. 2012; Blanco et al. 2016). This species has already developed resistance to 45 active ingredients which include pesticides and several Bt transgenic events (Mota-Sanchez and Wise 2023), which makes the control of this pest even more difficult.

Biological control plays an important role in integrated pest management in Bt crops because natural enemies can delay the evolution of insect resistance to this technology (Liu et al. 2014). Research interest in parasitoids of *Spodoptera* aiming at its control has been high for at least a century, especially for the fall armyworm (Molina-Ochoa et al. 2003). A few species of *Aleiodes* are recorded as parasitoids of *Spodoptera*, of which *Aleiodes laphygmae* (Viereck) is the most commonly reported, mainly in North America (USA and Mexico) (Yu et al. 2016). Overall, *Aleiodes* have been reported to have little impact on fall armyworm populations in crops, but its prevalence among parasitoids in grasses reached 92% (Braman et al. 2004). It is important to clarify that the record of *A. laphygmae* in Brazil, attributed to Cruz et al. (1997a, b) by Molina-Ochoa et al. (2003), is not correct, and other records from South America are sparse and may also be incorrect. Other species reared from *Spodoptera* include *Aleiodes terminalis* Creson in *S. frugiperda* and *S. ornithogalli*, in North America (Marsh and Shaw 2001), and *Aleiodes vaughani* (Muesebeck) in *S. frugiperda* and *S. eridania*, in Central and South America (Muesebeck 1960; Redolfi-Huiza and Marin-Loayza 1992; Shaw et al. 1997a, b). The discovery of a new species of *Aleiodes* parasitizing caterpillars of the *Spodoptera* complex in Brazil opens new prospects for the use of this parasitoid in biological control programs for *Spodoptera* species.

The objective of this paper is to describe a new species of the genus *Aleiodes*, with excellent potential for application in the biological control of *Spodoptera.* Because *Aleiodes* is a highly diverse genus, with hundreds of described species, we also discuss the placement of this new species in the context of the proposed subdivisions of the genus into species-groups, especially the *circumscriptus/gastritor* group and other similar or related groups. We also present an illustrated key to species based on morphological characters and provide DNA barcode sequences of the new species, aiming to facilitate easier identification of the new species, especially for applied entomologists (Shimbori et al. 2023). A summary of the biological information of the neotropical species previously included in the *circumscriptus/gastritor* group is also provided.

## Material and methods

### Sampling

Specimens of *Aleiodes ceres* Shimbori sp. n. were collected in 2016/2017 soybean (*Glycine max* (L.) Merrill) crop season in São José dos Pinhais (25°36′49.0″S 49°08′01″W), Paraná, Brazil. In the 2017/2018 and 2018/2019 soybean crop seasons, between January and March, a survey of pest caterpillars was conducted weekly by the beat cloth method (Shepard et al. 1974) in 10 points of two areas of genetically modified soybeans. One of the areas (non-Bt area) was planted with the seed variety NA5909RG, expressing tolerance to the herbicide glyphosate, during the two crop seasons evaluated. The other area (Bt area) was planted with the variety Syn13671 IPRO in the first crop season, and in the second crop season with variety M5917 IPRO and Syn1561 IPRO. Varieties from the Bt area also provide tolerance to the herbicide glyphosate, besides expressing the Cry1Ac protein of *Bacillus thuringiensis* (Bt) which confers resistance to target lepidopteran species.

Collected caterpillars were separated individually in plastic containers, identified at species level, and fed with soybean leaves from the same variety in which they were collected. Within the species collected, we identified the most common species of the *Spodoptera* complex in Brazil, namely *Spodoptera cosmioides* (Walker), *Spodoptera eridania* (Stoll), and *Spodoptera frugiperda* (J.E. Smith). The caterpillars were observed daily for parasitism. After emergence of the adult parasitoids, lab-reared *Spodoptera eridania* caterpillars were provided aiming to establish a laboratory rearing of the parasitoids. Specimens of the parasitoid were killed at low temperature (− 20 °C) and preserved in 99% ethanol for identification.

Additional samplings were conducted in the 2019 maize (*Zea mays* L.) crop season, in Pinhais (25°24′01″ S 49°07′01″ W), Paraná, Brazil, in an experimental area with the variety 30F53 Pioneer. The methodology used was similar to the soybean sampling.

### Biological data

Fifteen 48-h-old parasitoid females previously mated were kept individually in plastic containers (Æ 98.5 × 210 mm) with a drop of honey in an incubator room (25 ± 2 °C, 70 ± 10% UR and 14:10 photoperiod). Second instar larvae of *S. eridania* (*n* = 15) were provided for each female, and after 24 h, caterpillars were removed and new specimens were offered. This procedure was repeated for 3 days.

Larvae were individualized in plastic containers (500 mL) and daily, soybean leaves from the variety BRS 1003IPRO which express tolerance to the herbicide glyphosate and the Cry1Ac protein of Bt were provided. We evaluated the total number of larvae parasitized (by observing the formation of mummies), development time (in days), and emergence rate.

### Taxonomy

For identification of the subfamily Rogadinae (Braconidae), see van Achterberg (1993) or Sharkey (1997). For recognition of rogadine genera, refer to the identification keys of van Achterberg (1991) or Shaw (1997). The definition of *Aleiodes* adopted here follows that of van Achterberg (1991) and Shaw (2006). For identification of *gastritor* and *circumscriptus* species-groups, see Shaw et al. (1997a, b) and Fortier and Shaw (1999). The identification key presented here was adapted from the key to species of *Aleiodes* from Ecuador (Shimbori and Shaw 2014). Additional images for the majority of the species in the key can be found there.

Morphological terminology for descriptions follows that of Sharkey and Wharton (1997), S.R. Shaw et al. (1997a, b), Shimbori et al. (2015), Shimbori et al. (2016), and Garro et al. (2017). Microsculpture terminology follows that of Harris (1979). Wing veins terminology follows the system adopted by Sharkey and Wharton (1997). Measurements were taken following Shimbori et al. (2016). We follow Karlsson and Ronquist (2012) in defining the mesosomal area just lateral to the mesoscutellar disc (or scutellum) as the ‘‘mesoscutellar trough.” Abbreviations used throughout the descriptions are as follows:

- OOL: shortest distance between eye and lateral ocellus
- OD: maximum diameter of lateral ocellus
- POL: shortest distance between lateral ocelli
- T1: metasomal tergite 1
- T2: metasomal tergite 2
- T3: metasomal tergite 3.

Examined specimens are deposited in the following collections:

- Coleção Entomológica do Departamento de Ecologia e Biologia Evolutiva da Universidade Federal de São Carlos, São Carlos, Brazil (DCBU)
- Coleção Entomológica Padre Jesus S. Moure, Departamento de Zoologia da Universidade Federal do Paraná, Curitiba, Brazil (DZUP)
- University of Wyoming Insect Museum, Department of Ecosystem Science and Management, University of Wyoming, Laramie, WY, USA (UWIM)

### Molecular methods

Genomic DNA was extracted from the whole wasp using DNeasy Blood & Tissue Kit (QIAGEN Inc., Valencia, California) following the manufacturer’s instructions. Subsequent DNA purification was performed by Ethanol Precipitation (Sambrook and Russell 2001) and then DNA extracts were resuspended in 50 µL of TE Buffer. The COI fragment belonging to the barcoding locus was amplified using the universal LCO 1490 and HCO 2198 primers (Folmer et al. 1994). Polymerase chain reaction was carried in 25 µL final volume (2.5 mM MgCl_2_, 0.2 mM dNTP Mix, 0.2 µM each primer, 1 × HOT FIREPol® Buffer B2, and 1 U HOT FIREPol® DNA Polymerase, Solis Biodyne) and followed a cycling process of initial denaturation at 95°C for 15 min; 35 cycles of denaturation at 95°C for 45 s, annealing at 56°C for 30 s, and extension at 72°C for 1 min; and finally a final extension at 72°C for 5 min. PCR products were purified by polyethylene glycol precipitation (Lis and Schleif 1975) and sequenced at Macrogen, Seoul, South Korea.

### Phylogeny

The molecular dataset for phylogenetic analyses was mainly gathered from GenBank and BOLD, based on previous publications (Areekul-Butcher et al. 2012; Shimbori and Shaw 2014; van Achterberg et al. 2020; Sharkey et al. 2021), aiming to include representatives of most species-groups in the genus *Aleiodes*, and all biogeographic regions. A total of 286 sequences were used for the analyses, four of them of species of *Heterogamus* (Appendix 1 in Supplementary Information (SI)). Sequences were aligned using MAFFT v. 7.130b (Katoh and Standley 2013). Molecular phylogeny was performed using IQ-TREE v 1.6.12 maximum-likelihood analyses (Nguyen et al. 2015), with ultrafast bootstrap (6000 replicates) (Hoang et al. 2018). The ModelFinder (Kalyaanamoorthy et al. 2017) was used to choose the best model for each of the three partitions, corresponding to each codon position (Chernomor et al. 2016). The best-fit models according to Bayesian information criterion were TIM2 + F + R6 for partition 1 and TIM + F + I + G4 for partition 2 + 3. A single branch SH-aLRT test was performed with 2000 replicates (Guindon et al. 2010).

**Fig. 1:**
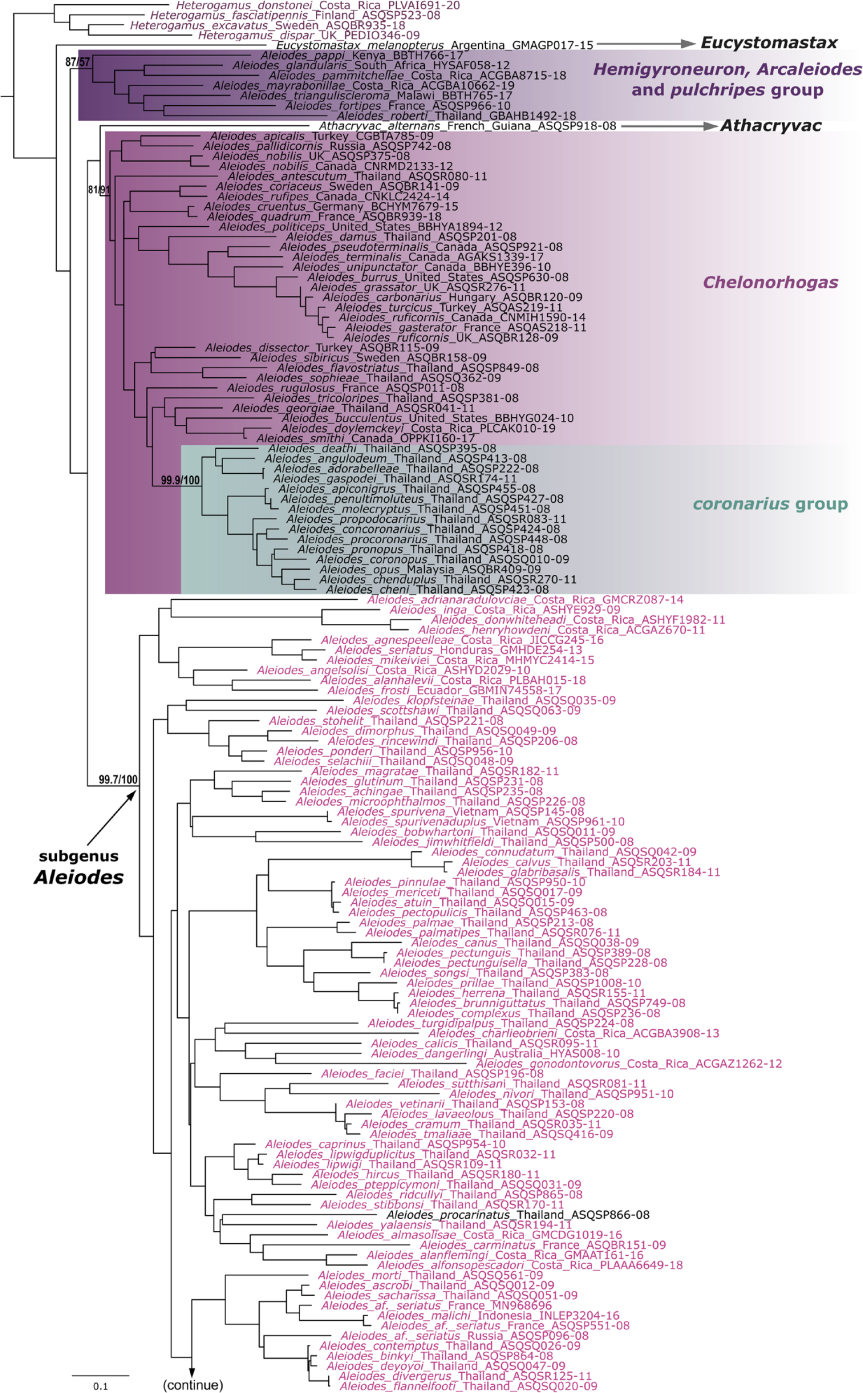

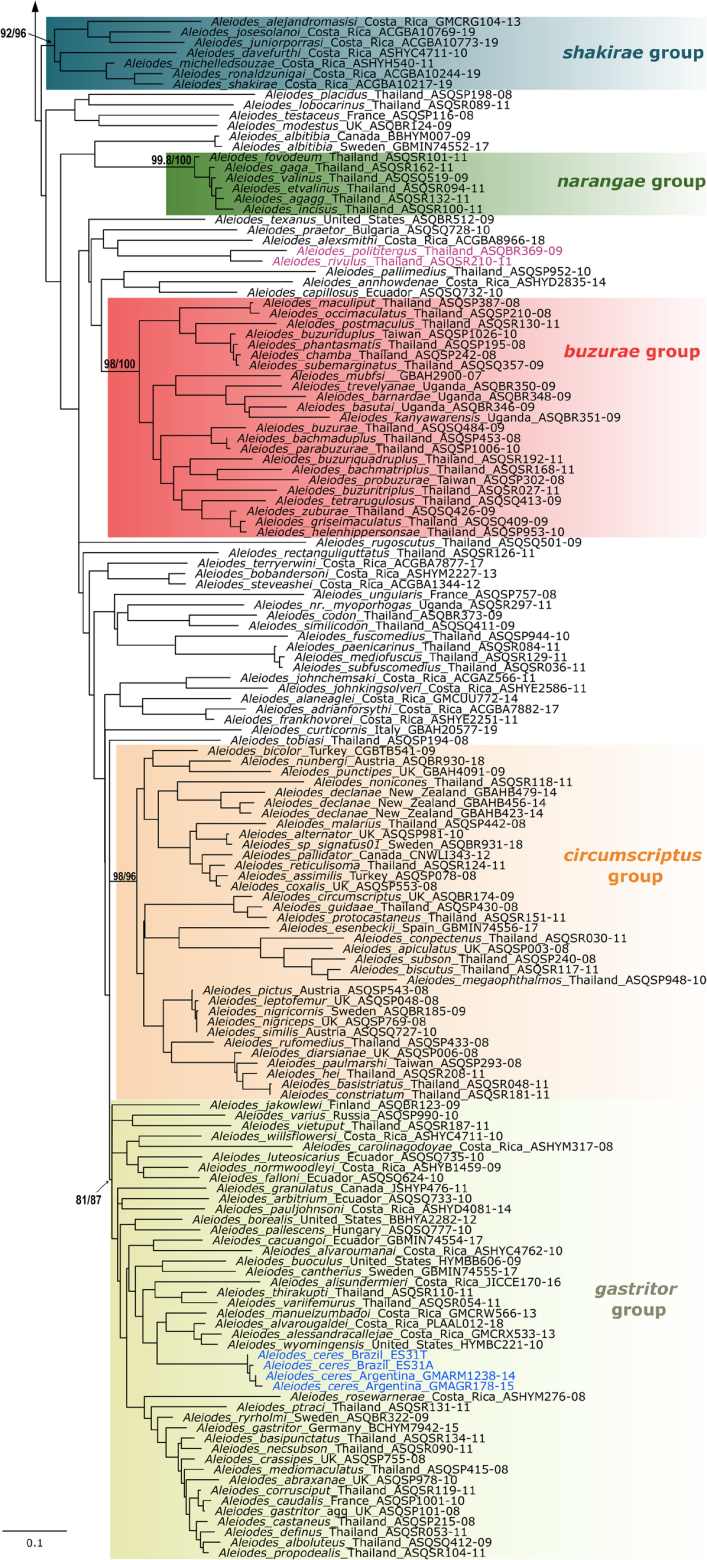
**a**, **b** Maximum likelihood tree based on DNA barcode sequence data for the genus *Aleiodes.* Clades corresponding to species-groups and subgenera proposed by several authors are highlighted. Terminal taxa with the comb of specialized flattened setae at the apex of the hind tibia are colored purple, and of the new species, *Aleiodes ceres*, are colored blue. Terminal text shows species name, voucher code, and country of provenance. Support values, SH-aLRT support (%)/ultrafast bootstrap support (%), are shown in relevant clades

The resulting best tree is presented with species-groups highlighted with the same color code used in van Achterberg et al. (2020) and Areekul-Butcher et al. (2012) for easier comparison. Our results are in great conformity with molecular trees for Thai (Areekul-Butcher et al. 2012) and Palaearctic *Aleiodes* (van Achterberg et al. 2020), all based on the COI Barcode locus, although the subgenus *Chelonorhogas* (or the *apicalis* species-group) is recovered as a monophyletic clade rather than a grade leading to the subgenus *Aleiodes* (Fig. 1).

Phylogenies based on a single locus, such as the one presented here, have serious limitations and are to be taken as a tentative assessment of the phylogenetic relationships. The main objectives of the analysis are to aid species delimitation and support the discussion on the classification of the new species at the species-group level, including more elements of the neotropical fauna. Relevant results for the systematics of neotropical *Aleiodes* are the recognition of a relatively basal clade within subgenus *Aleiodes*, which is morphologically similar to the *gastritor* Thunberg species-group, and the absence of the *circumscriptus* Nees*/bicolor* Spinola group in the New World. Additionally, several neotropical species are found scattered within the *gastritor* clade, including *A. luteosicarius* Shimbori & Shaw, here transferred from *pallidator* Thunberg to *gastritor*, and the new species *A. ceres* sp. n.

## Results

### Comments on the *gastritor*, *circumscriptus*, and similar species-groups in the subgenus *Aleiodes* in the Neotropical region

As proposed by Shaw et al. (1997a, b) and Fortier and Shaw (1999), the *gastritor* species-group would be distinguished from the *circumscriptus* species-group in having relatively larger ocelli (OD > OOL), and metasomal terga usually yellow (although a pattern similar to that of *circumscriptus* is possible), as opposed to the smaller ocelli (OD < OOL) and metasomal terga always yellow medially and black laterally in *circumscriptus*. In addition, the *circumscriptus* group is broadly associated with Noctuoidea hosts, while *gastritor* group species are mostly associated and Geometridae (Table 1, Fig. 5 in Zaldívar-Riverón et al. 2008 and Fig. 7 in van Achterberg et al. 2020). However, as noted by Townsend and Shaw (2009), several (borderline) Neotropical species do not precisely fit in the diagnoses of the *gastritor* or *circumscriptus* species-groups*.* These authors used this evidence to propose the *circumscriptus/gastritor* species-group, lumping the two groups.

**Table 1:** Distribution, biological associations, and species-groups of the Neotropical species of the subgenus *Aleiodes* Wesmael, 1838, related to the *gastritor* species-group. The biological information is based on Townsend and Shaw (2009), Abreu et al. (2014), Shimbori and Shaw (2014), Yu et al. (2016), and Sharkey et al. (2021)

Species	Distribution	Host Species	Host family	Plant	Plant family
*gastritor* species-group					
*aclydis* Townsend	Ecuador		Geometridae	*Ocotea*	Lauraceae
*albigena* Shimbori & Shaw	Ecuador	nr. *Desmotricha*	Erebidae	*Chusquea scandens*	Poaceae
*albiterminus* Townsend	Ecuador		Geometridae	*Alnus acuminata*	Betulaceae
*alessandracallejae* Sharkey	Costa Rica				
*alisundermieri* Sharkey	Costa Rica				
*alvarougaldei* Sharkey	Costa Rica				
*alvaroumanai* Sharkey	Costa Rica	*Syngamia florella*	Crambridae	*Spermacoce exilis*	Rubiaceae
*arbitrium* Townsend	Ecuador	*Psaliodes castanea*	Geometridae	*Diplazium costale*; *Dennstaedtia cornuta*	Dryopteridadceae; Dennstaedtiaceae
*argentiniensis* Shimbori & Martinez	Argentina				
*atripileatus* Townsend	Ecuador	*Hypena* sp.	Noctuidae	*Phenax rugosus*, *Boemeria bullata*, *Miriocarpa* sp.	Urticaceae
*bimaculatus* Shimbori & Shaw	Ecuador				
*bonariensis* (Brèthes)	Argentina				
*cacuangoi* Shimbori & Shaw	Ecuador	“linea blanca en la espalda chusquea”	Geometridae	*C. scandens*	Poaceae
*ceres* **sp. n**	Argentina; Brazil	*Spodoptera cosmioides*, *S. eridania*; *S. frugiperda*	Noctuidae	*Glycine max*; *Zea mays*	Fabaceae Poaceae
*falloni* Shimbori & Shaw	Ecuador				
*gossypii* (Muesebeck)	Brazil; Colombia; Peru; Venezuela	*Alabama argilacea*; *Anomis* sp.	Erebidae	*Gossypium* sp.	Malvaceae
*laphygmae* (Viereck)	North and Central America	*Mythimna unipuncta*, *Spodoptera* spp.	Noctuidae	Many	
*leptocarina* Fortier	Costa Rica	*Dysschema viuda*	Erebidae	*Zanthoxylum riedelianum*	Rutaceae
*luteosicarius* Shimbori & Shaw	Ecuador				
*manuelzumbadoi* Sharkey	Costa Rica				
*manuelzumbadoi* Sharkey	Costa Rica				
*mirandae* Shimbori & Shaw	Ecuador	“palito café chusquea”	Geometridae	*C. scandens*	Poaceae
*napo* Shimbori & Shaw	Ecuador	“raya roja a los lados chusquea”	Noctuidae	*C. scandens*	Poaceae
*nubicola* Shimbori & Shaw	Ecuador	“palito café chusquea”	Geometridae	*C. scandens*	Poaceae
*onyx* Shimbori & Shaw	Ecuador	“espalda tomate rubiacea”	Zygaenidae	*Notopleura plagiantha*	Rubiaceae
*pauljohnsoni* Sharkey	Costa Rica	*Erosia veninotata*	Uraniidae	*Randia grandifolia*	Rubiaceae
*yanayacu* Shimbori & Shaw	Ecuador		Geometridae	*Phenax rugosus*	Urticaceae
*shakirae* species-group					
*alejandromasisi* Sharkey	Costa Rica				
*davefurthi* Sharkey	Costa Rica	*Herbita medona*	Geometridae	*Hirtella racemosa*	Chrysobalanaceae
*japi* Shimbori & Penteado-Dias	Brazil	*Physocleora grosica*; *Ischnopteris* sp.	Geometridae	*Alchornea triplinervia*	Euphorbiaceae
*michelledsouzae* Sharkey	Costa Rica	Yidalpta auragali*	Erebidae*	*Securidaca sylvestris*	Polygalaceae
*shakirae* Shimbori & Shaw	Ecuador	“palito café chusquea”	Geometridae	*C. scandens*	Poaceae
*speciosus* Townsend	Ecuador			*Miconia* sp.	Melastomataceae
*townsendi* Shimbori & Shaw	Ecuador		Geometridae	*Dendrophobium lloense*	Asteraceae
Species-group not assigned					
*kingmani* Shimbori & Shaw	Ecuador		Geometridae	*C. scandens*	Poaceae
*tzantza* Shimbori & Shaw	Ecuador		Noctuidae		

^*^Host record for *A. michelledsouzae* is based on a mummy collected on the referred plant (Voucher Code: 11-SRNP-31492; Parasite code: DHJPAR0042782: “encontrado con momia de parásito silvestre en estadío intermedio entre PU y U, tome fotos de la momia”). Images of the mummy (available at http://janzen.sas.upenn.edu/caterpillars/database.lasso) indicate it is most likely from a Geometridae caterpillar

Both *gastritor* and *circumscriptus* species-groups are recovered as derived groups in the subgenus *Aleiodes* (sensu Zaldívar-Riverón et al. 2008), sister to one another, in all molecular phylogenies published so far (Zaldívar-Riverón et al. 2008; Areekul-Butcher et al. 2012; van Achterberg et al. 2020). Thus, the classification proposed by Townsend and Shaw (2009) could reflect the evolutionary history of *Aleiodes*, comprising the most derived clade in the subgenus *Aleiodes*. In this broader sense, the *circumscriptus/gastritor* group would include species of other small groups such as *pallidator*, *compressor* Herrich-Schaffer, and *coxalis* Spinola (= *bicolor* and excluding *Tetrasphaeropix* Ashmead), and many unassigned species (see trees in Zaldívar-Riverón et al. 2008 and van Achterberg et al. 2020).

There are two problems in using this broader concept. First, the higher morphological and biological variation makes it difficult to diagnose the group. The *circumscriptus* group, for instance, is morphologically distinct and nearly entirely associated with one host family, the Noctuidae. This group is also absent in the New World, whereas the *gastritor* species-group is widespread. Second, the Neotropical species that would fit in the broader morphological concept do not form a monophyletic clade. Instead, at least two clades comprise Neotropical species, one being the *gastritor* and a second and relatively basal clade within the subgenus *Aleiodes*, which we refer to as the *shakirae* Shimbori & Shaw clade*.* This basal clade comprises Neotropical species with geometrid hosts and “mixed” characters, of both the *gastritor* and *circumscriptus* groups (Fig. 1). Since our results are preliminary with respect to the phylogenetic history of the group, and the key point of separating species-groups is to facilitate the identification of this massive genus, we consider these subdivisions useful for their purpose of separating the genus into manageable parts, as long as each part is identifiable, either using morphology or DNA.

Subdivisions of this large clade already exist (e.g., groups *bicolor/circumscriptus*, *gastritor*, and *similis* Curtis in the Palearctic—van Achterberg et al. 2020), as well as within the subgenus *Aleiodes* as a whole (e.g., the *buzurae* He & Chen, *Tetrasphaeropyx* and *risaae* Quicke & Butcher groups—van Achterberg et al. 2020; Fortier 2006, 2009; Quicke et al. 2006; Zaldívar-Riverón et al. 2008; Areekul-Butcher et al. 2012), and are desirable as a way to define smaller monophyletic groups, facilitating identifications and revisions. However, considering the morphological variation in the Neotropical species, it is clear that the main characters used to distinguish *gastritor* and *circumscriptus* are homoplasious. In fact, nearly all Neotropical species in the subgenus *Aleiodes*,^1^ excluding the species in the *seriatus* group, will fit in the broader morphological concept of *circumscriptus/gastritor.* For instance, *A. kingmani* Shimbori & Shaw is not distinct from other *gastritor* group species, but the morphology of its mummy resembles the suspended mummies of the *buzurae* group (Quicke et al. 2006; Shimbori and Shaw 2014). Besides mummy shape, species in the *buzurae* group are morphologically similar to *gastritor*, being distinguished from it by the strongly sculptured fourth metasomal terga (Quicke et al. 2006), a feature absent in described Neotropical species. Our phylogeny recovers neotropical species of the subgenus *Aleiodes* scattered in several parts of the tree, for most of which we do not have morphological information (Sharkey et al. 2021). Therefore, further proposals for subdivisions are difficult and of limited value without a refined and comprehensive phylogenetic study of the genus.

Based on the evidence discussed above, we summarize the following conclusions on the systematics of the *Aleiodes* species-groups:

- The ***circumscriptus*** species-group (including the *bicolor* or *coxalis* species-group and *A. pallidator* species-group) is likely a monophyletic group absent in the New World (it may also be called *bicolor* or *coxalis*—priority of Spinola 1808 over Nees 1834). It should be treated as its own group, as proposed by van Achterberg et al. (2020).
- Regarding the ***pallidator*** group, the type species *Aleiodes pallidator* is recovered within the Palearctic *circumscriptus* + *bicolor* + *coxalis* clade (van Achterberg et al. 2020; Fig. 1). All species in this clade, including *A. pallidator*, are native to the Old World (Shaw et al. 2006), even though *A. pallidator* was introduced from Europe to North America for biological control (Shaw 2006). In addition, *A. pallidator* is the only known species in the *pallidator* species-group, sensu Shaw et al. (2013), without a distinct pecten in the tarsal claws. A closer relationship with *A. coxalis* was also recovered in a phylogeny using COI + 28S (Zaldívar-Riverón et al. 2008). If the phylogenetic inferences are correct, *pallidator* should be part of the *circumscriptus* (= *bicolor*) group, and if the biogeographical distribution of *circumscriptus* is indeed restricted to the Old World, the species from the New World are better placed in another group. The homoplasious nature of the characters defining *pallidator* seems corroborated by all available evidence, and by the fact that the only described neotropical species in the *pallidator* group, *A. luteosicarius*, is recovered in the *gastritor* group in our phylogeny (Fig. 1). Therefore, *A. luteosicarius* is here transferred to the *gastritor* species-group.
- The ***gastritor*** group is represented by many species in the New World, most of them undescribed. Currently, it is not possible to recognize this group using morphology alone, but our phylogeny indicates that neotropical species with morphological features of *gastritor* or *circumscriptus* (as originally recognized by Shaw et al. 1997a, b) in most cases belong to this clade.
- A relatively basal clade within the subgenus *Aleiodes*, comprising neotropical species with geometrid hosts and “mixed” characters of *gastritor* and *circumscriptus* (i.e., variable color and relatively large ocelli) was recovered and should be considered for a possible subdivision of the subgenus. Here, we provisionally name this clade the ***shakirae*** group, and include species with similar morphology or DNA barcode. This group is not yet distinguishable from neotropical members of the *gastritor* group; however, all species have larger eyes and ocelli, the ocelli-ocular distance shorter than the diameter of lateral ocelli, and are parasitoids on Geometridae. The metasoma is at least partly dark brown or black in described species. Species without morphological descriptions (Sharkey et al. 2021) are included based on our molecular phylogeny. According to images provided by Sharkey et al. (2021), some of those species have the body entirely honey yellow and therefore would not be keyed correctly to the *shakirae* group in the key provided below. The group is proposed to acknowledge the existence of this clade, even though morphological diagnosis and monophyly based on molecular data are preliminary.

### Key scope

The key presented below includes only neotropical species in the subgenus *Aleiodes*, previously in the *circumscriptus/gastritor* (Townsend and Shaw 2009) or *pallidator* species-groups (Shaw et al. 2013), and currently in one of the three groups listed below ((1) *gastritor*, (2) *shakirae*, or (3) unassigned)*.* These are neotropical species that would key in the *gastritor* and/or *circumscriptus* groups following the key provided by Shaw et al. (1997a, b) (couplet 16), or in the *pallidator* group (couplet 10), herein included in the *gastritor* group. These species do not comprise a monophyletic group but are recognizable by the following set of characters:

- *Diagnosis*. Apex of hind tibia without comb of specialized adpressed setae; hind wing vein RS slightly sinuate and enclosing a marginal cell which is narrowest around its middle length; metasomal tergites 1–3 with finely rugose or rugose costate sculpturing, never smooth or strongly costate; tarsal claws without distinct blackish pecten, but sometimes a yellowish pecten with thinner spines present; metasomal tergite 2 without a distinct smooth triangular area medio-basally; ocelli size variable.

The species included in the key are, therefore, members of at least two separate clades, for which a morphological distinction is currently unavailable, also including species that are not assigned to any of the two species-groups:

- 1. *Neotropical species included in the gastritor species-group*: As defined here, the group is one of the most species-rich in the Neotropics, comprising 20 species, namely *aclydis* Townsend, 2009; *albigena* Shimbori and Shaw 2014; *albiterminus* Townsend, 2009; *arbitrium* Townsend, 2009; *argentiniensis* Shimbori  & Martinez 2016; *atripileatus* Townsend, 2009; *bimaculatus* Shimbori & Shaw 2014; *bonariensis* (Brèthes 1910); *cacuangoi* Shimbori & Shaw 2014; *falloni* Shimbori & Shaw 2014; *gossypii* (Muesebeck 1960); *laphygmae* (Viereck 1912); *leptocarina* Fortier 2000; *luteosicarius* Shimbori & Shaw 2014; *mirandae* Shimbori & Shaw 2014; *napo* Shimbori & Shaw 2014; *nubicola* Shimbori & Shaw 2014; *onyx* Shimbori & Shaw 2014; *yanayacu* Shimbori & Shaw 2014; and *ceres* Shimbori sp. n.
- 2.* Species included in the Neotropical shakirae species-group*: based on morphology—*shakirae* Shimbori & Shaw 2014; *speciosus* Townsend, 2009; *townsendi* Shimbori & Shaw 2014; *japi* Shimbori & Penteado-Dias 2014; based on DNA barcode: *alejandromasisi* Sharkey 2021; *michelledsouzae* Sharkey 2021; *davefurthi* Sharkey 2021.
- 3.* Neotropical species in the subgenus Aleiodes not assigned to a group*: *kingmani* Shimbori & Shaw 2014; *tzantza* Shimbori & Shaw 2014. Both species are morphologically compatible with the *shakirae* species-group; however, *kingmani* is not assigned to *shakirae* based on mummy morphology, resembling the *buzurae* group, and *tzantza* because it is a parasitoid of Noctuidae, whereas all species in the *shakirae* species-group are parasitoids of Geometridae.

### Key to Neotropical species of *Aleiodes *in the *gastritor *and *shakirae *species-groups (*Aleiodes bonariensis* is not included due to poor condition of type specimen; species named by Sharkey et al. (2021) based only on molecular data are not included)

**Fig. 2:**
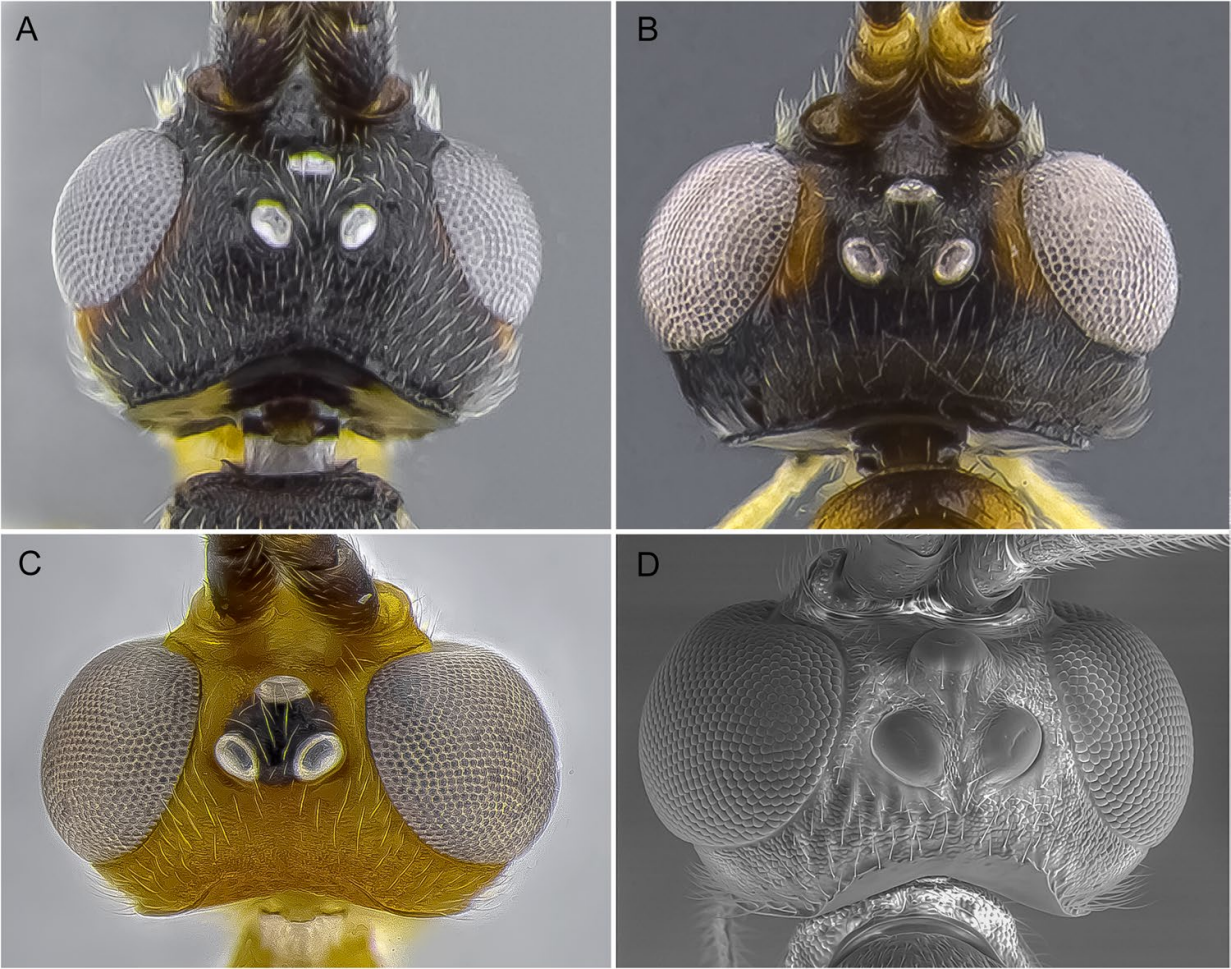
Head, dorsal. **A** *Aleiodes nubicola*; **B** *Aleiodes arbitrium*; **C** *Aleiodes shakirae*; **D** *Aleiodes japi*

**Fig. 3:**
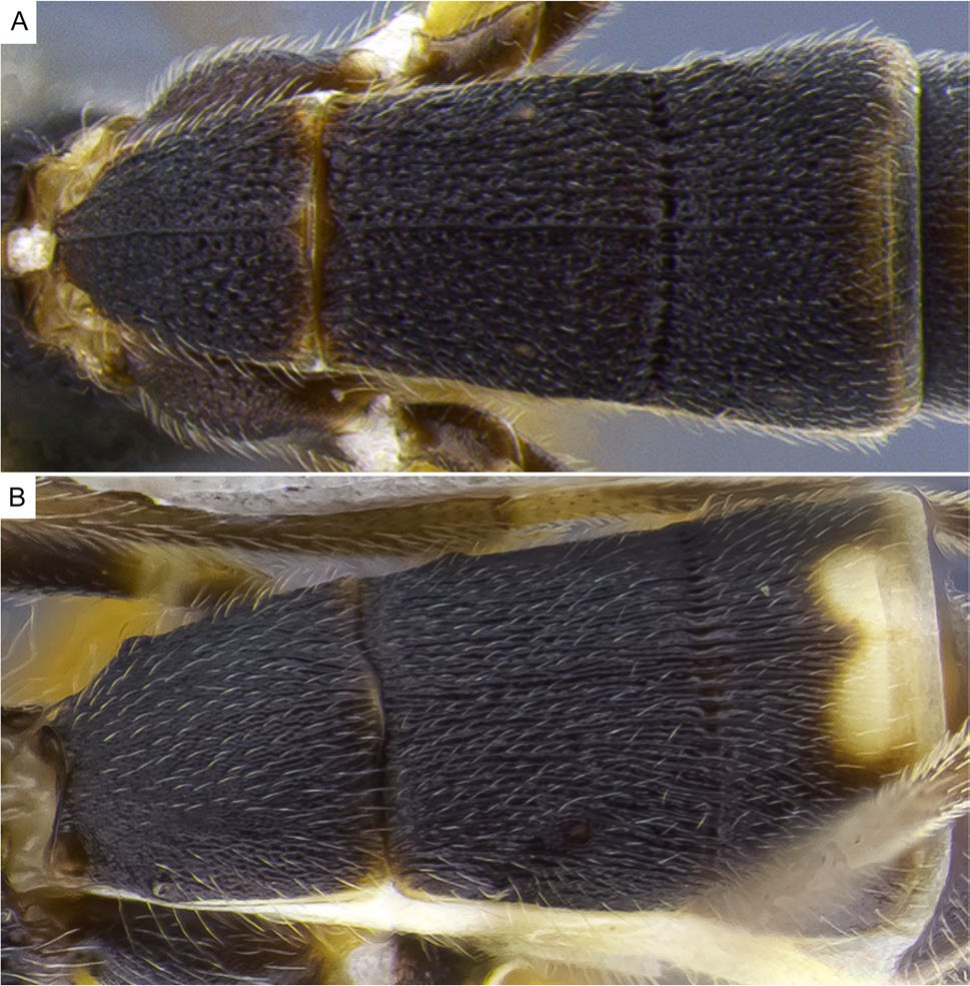
Metasoma, dorsal. **A** *Aleiodes nubicola*; **B** *Aleiodes albiterminus*

**Fig. 4:**
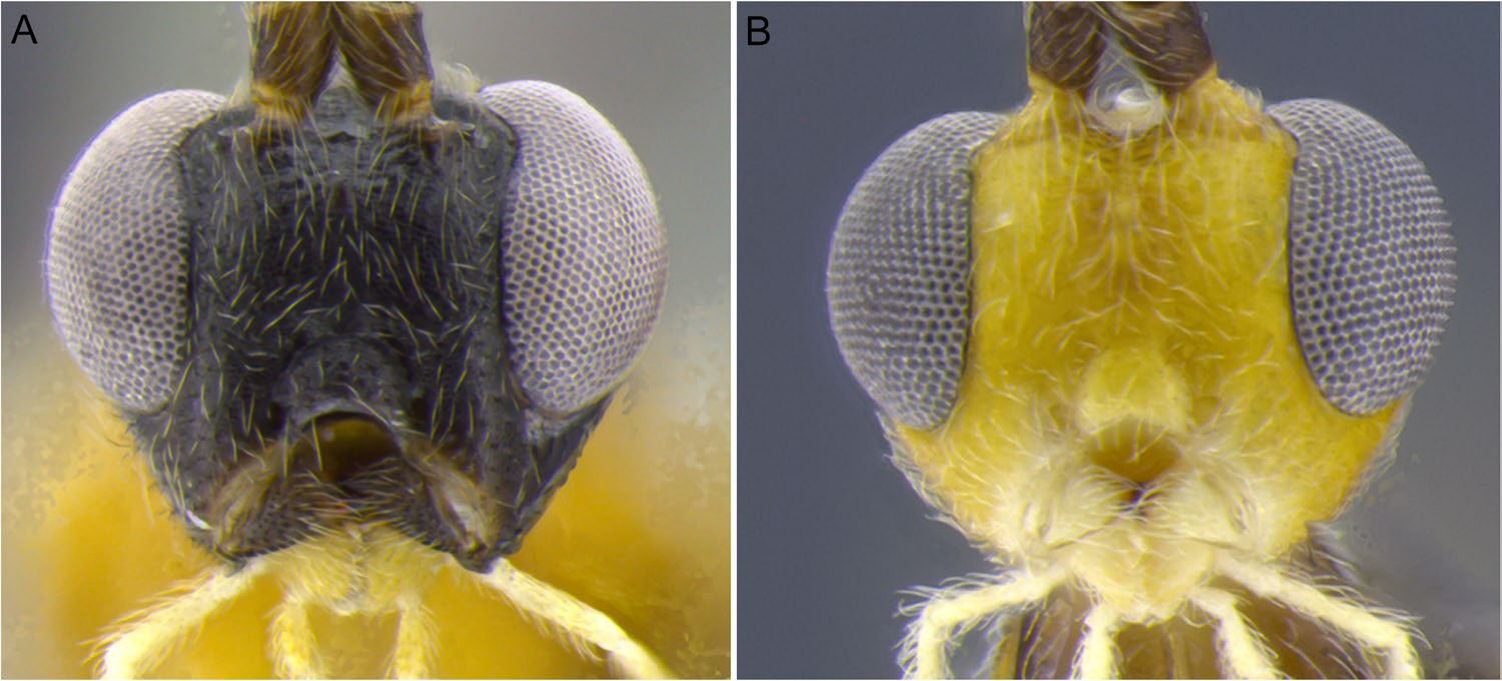
Face. **A** *Aleiodes arbitrium*; **B** *Aleiodes atripileatus*

**Fig. 5:**
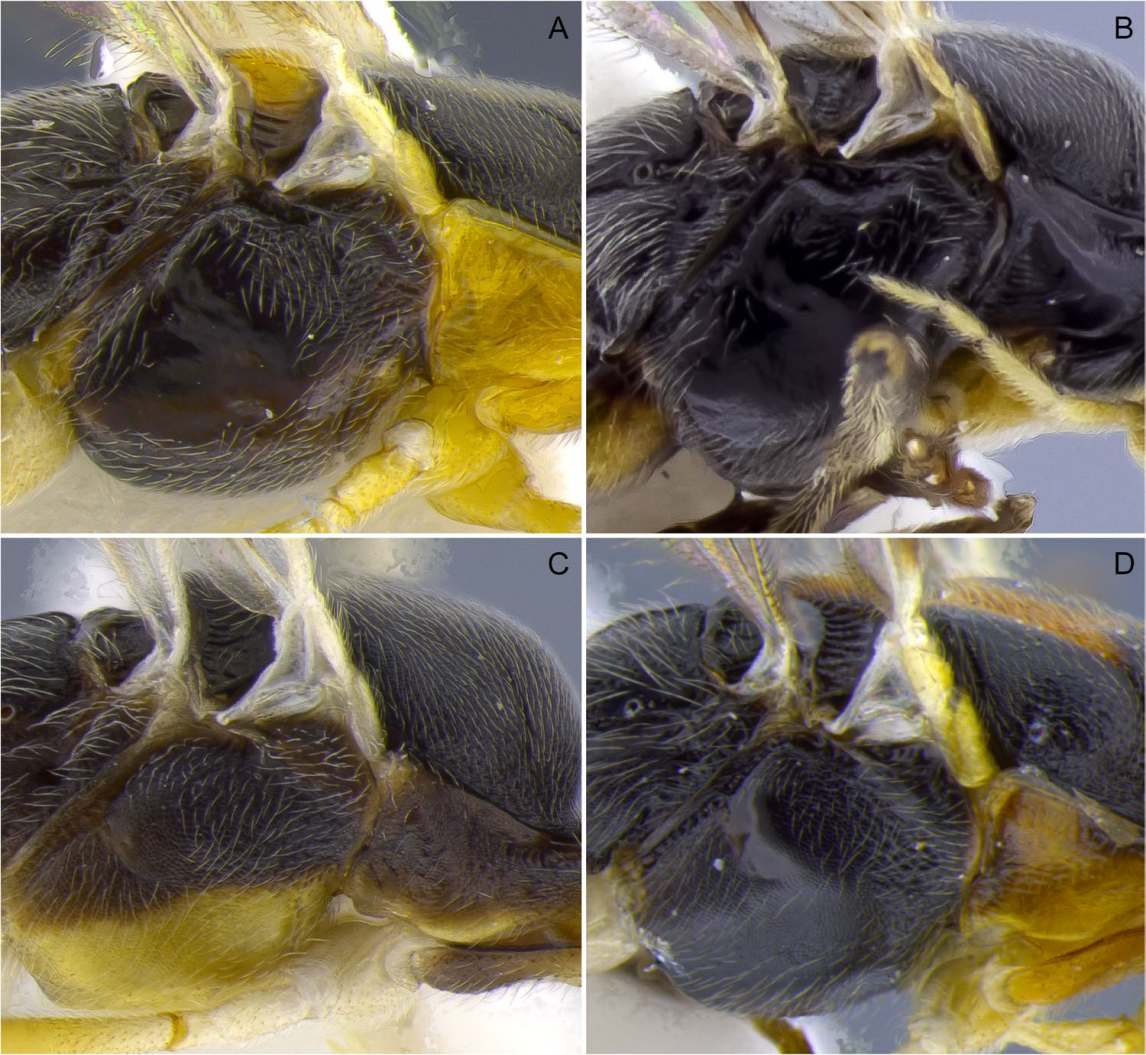
Mesosoma, lateral; detail of mesopleuron. **A** *Aleiodes mirandae*; **B** *Aleiodes napo*; **C** *Aleiodes atripileatus*; **D** *Aleiodes onyx*

**Fig. 6:**
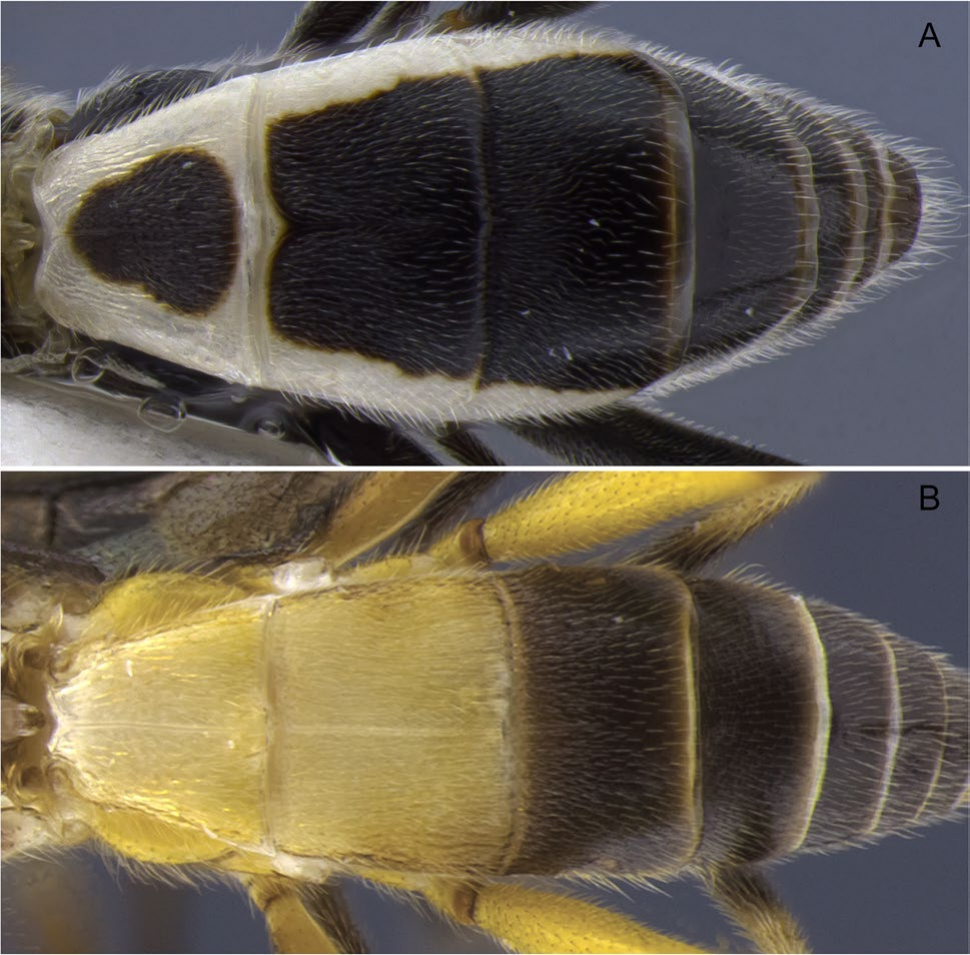
Metasoma, dorsal. **A** *Aleiodes napo*; **B** *Aleiodes yanayacu*

**Fig. 7:**
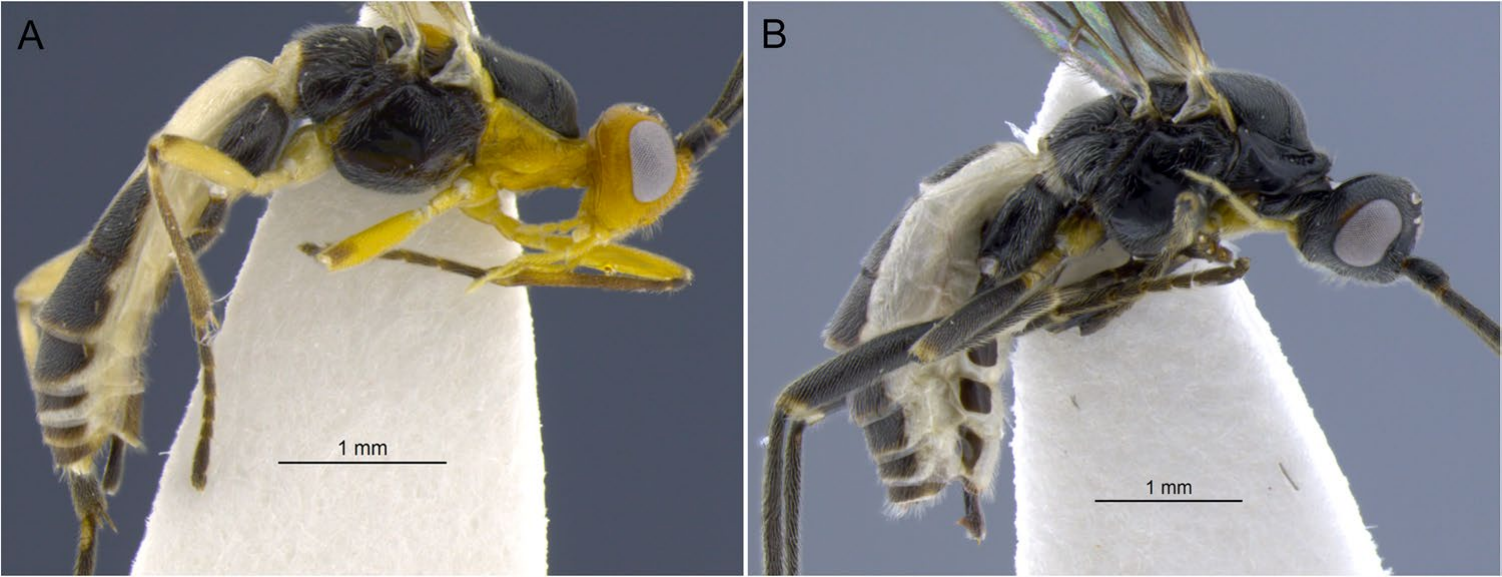
Habitus, lateral. **A** *Aleiodes mirandae*; **B** *Aleiodes napo*

**Fig. 8:**
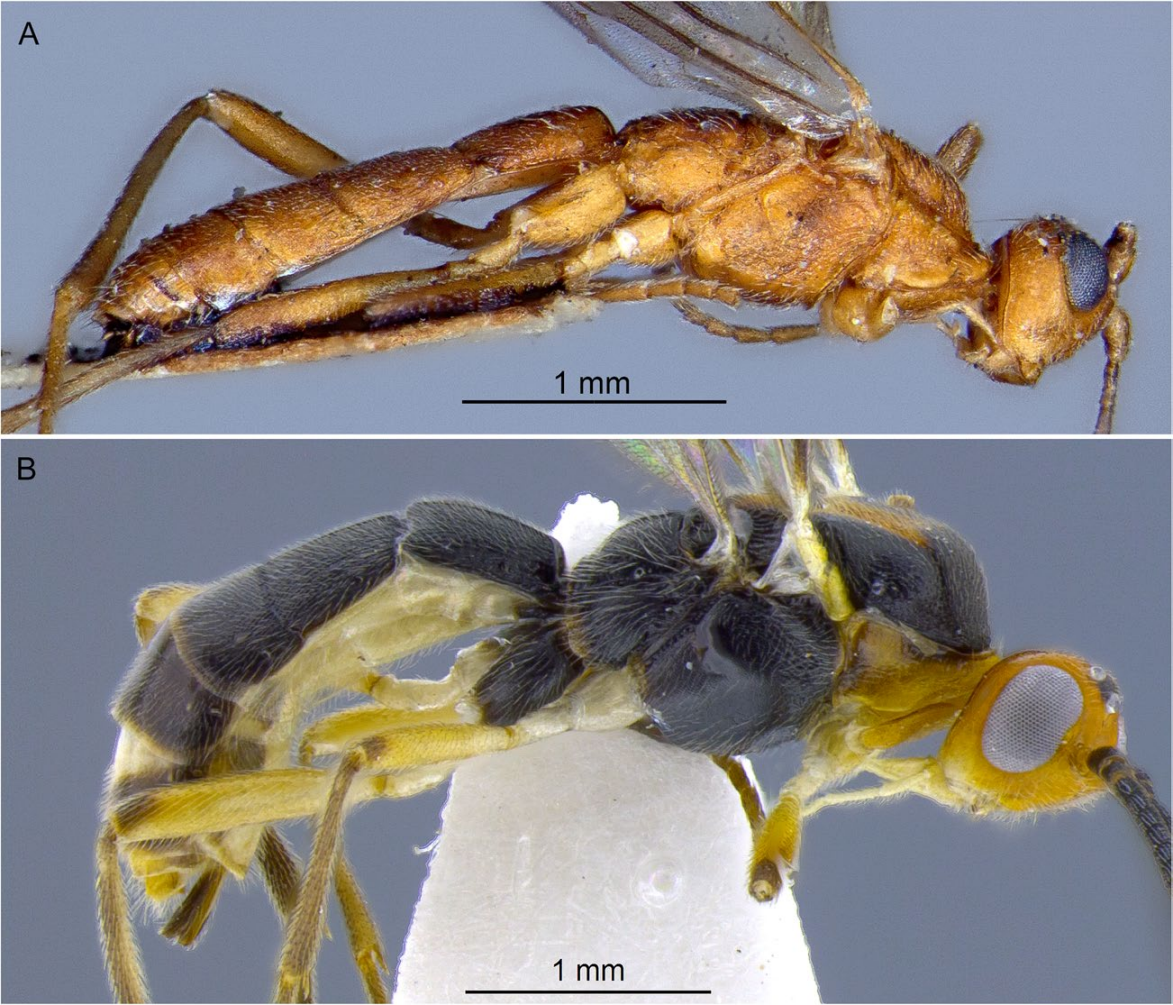
Habitus, lateral. **A** *Aleiodes argentiniensis*; **B** *Aleiodes onyx*

**Fig. 9:**
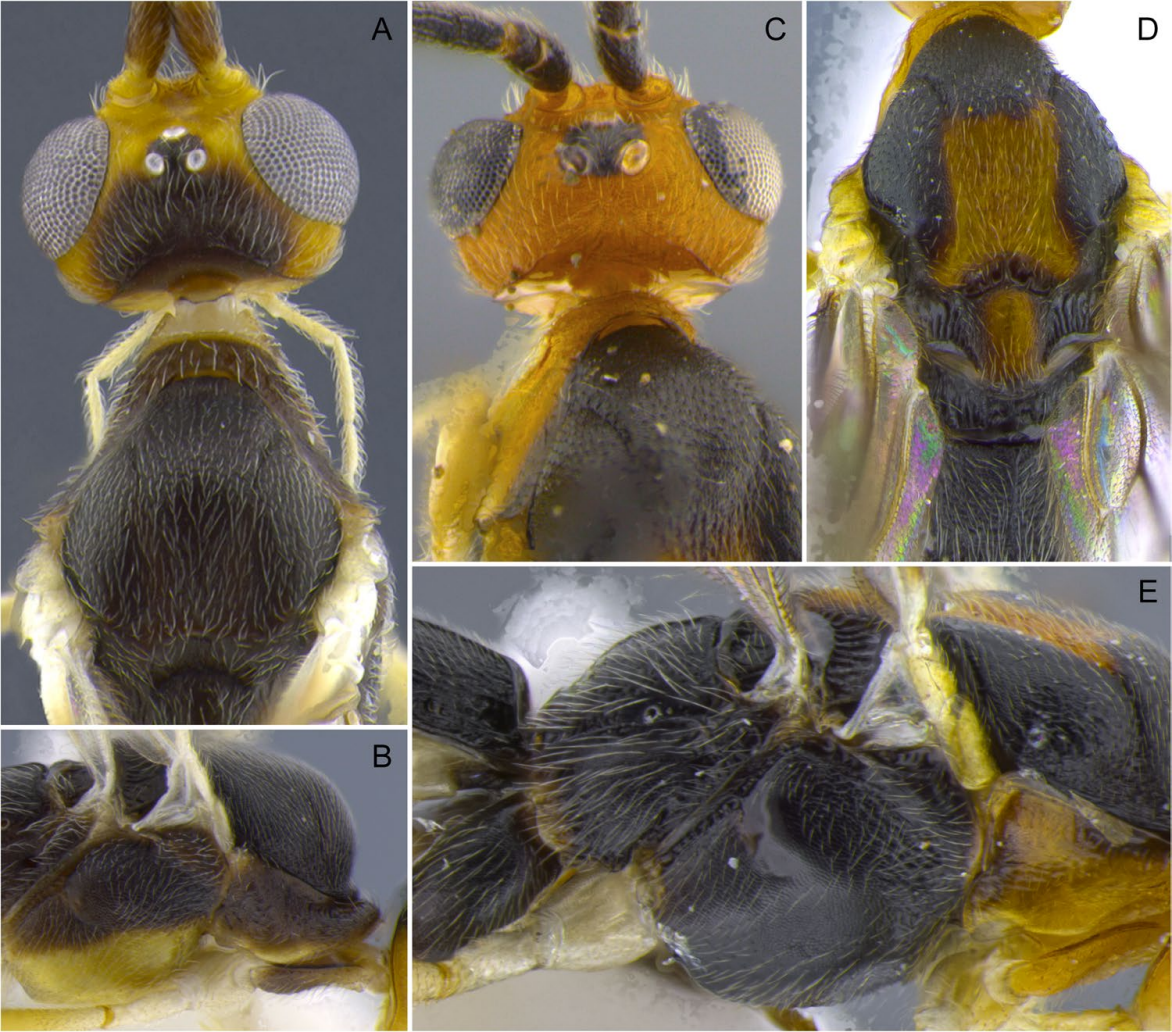
**A**, **B** *Aleiodes atripileatus*; **C**, **D**, **E** *Aleiodes onyx*

**Fig. 10:**
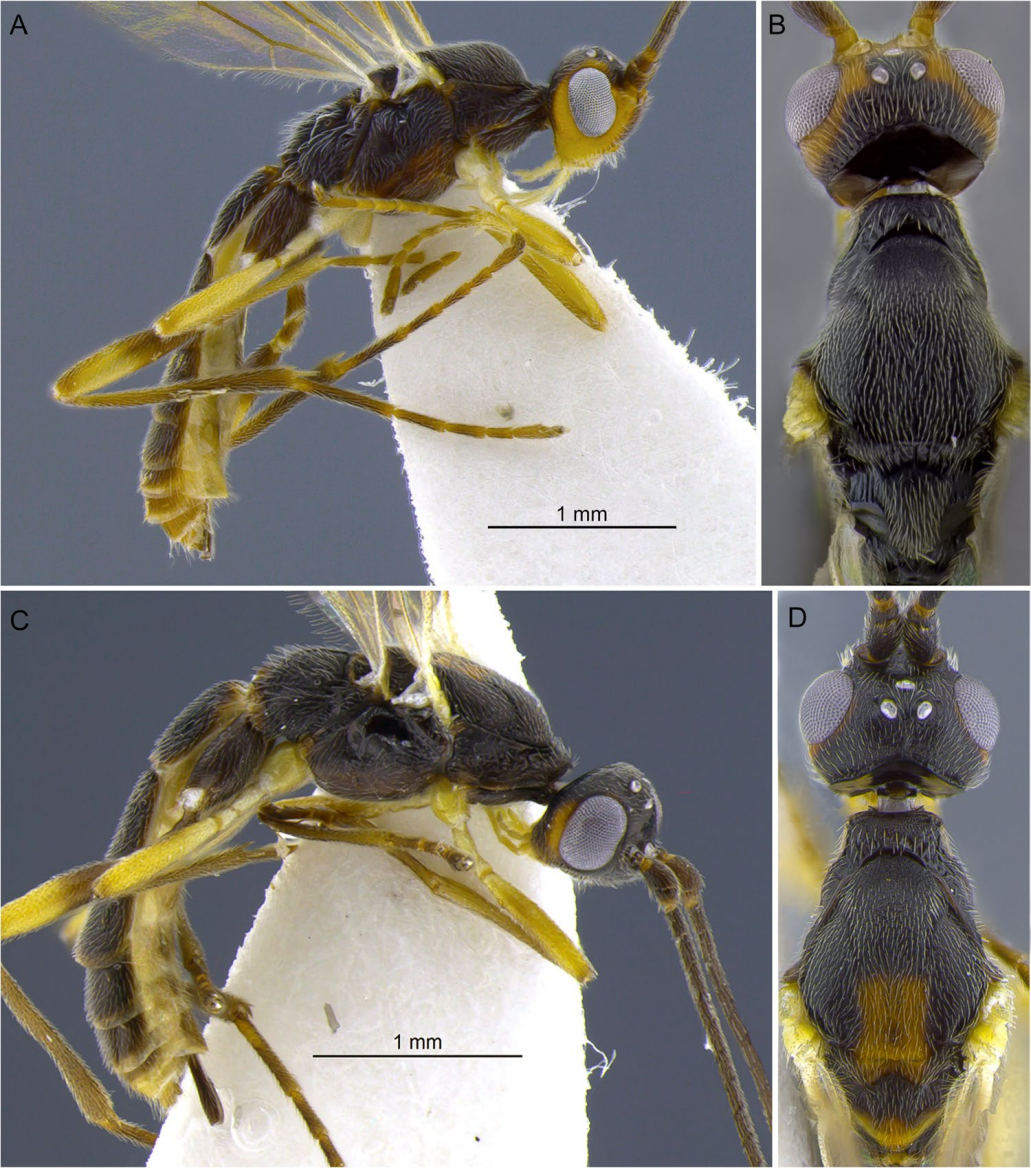
**A**, **B** *Aleiodes cacuangoi*; **C**, **D** *Aleiodes nubicola*

**Fig. 11:**
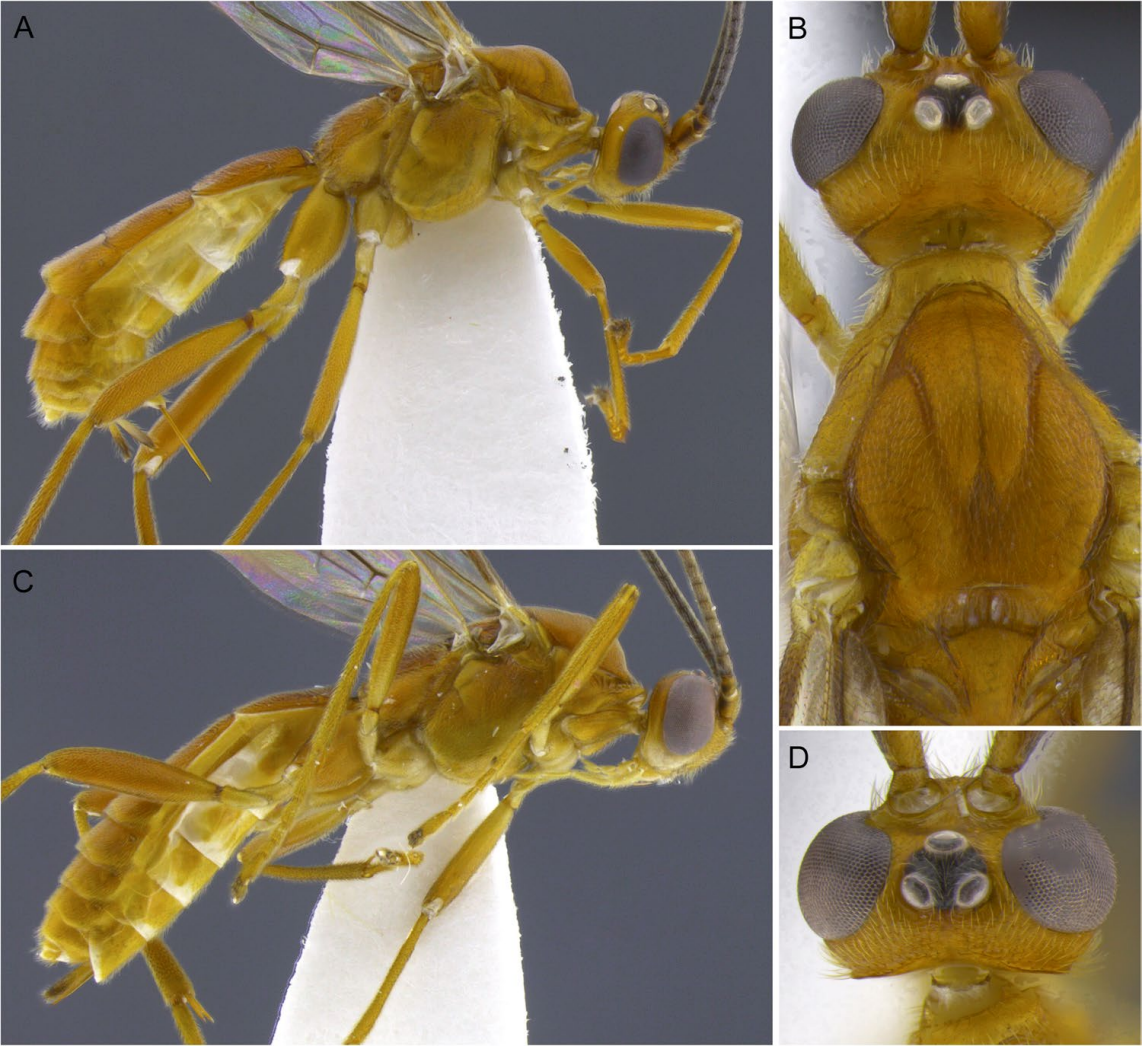
**A**, **B** *Aleiodes falloni*; **C**, **D** *Aleiodes luteosicarius*

**Fig. 12:**
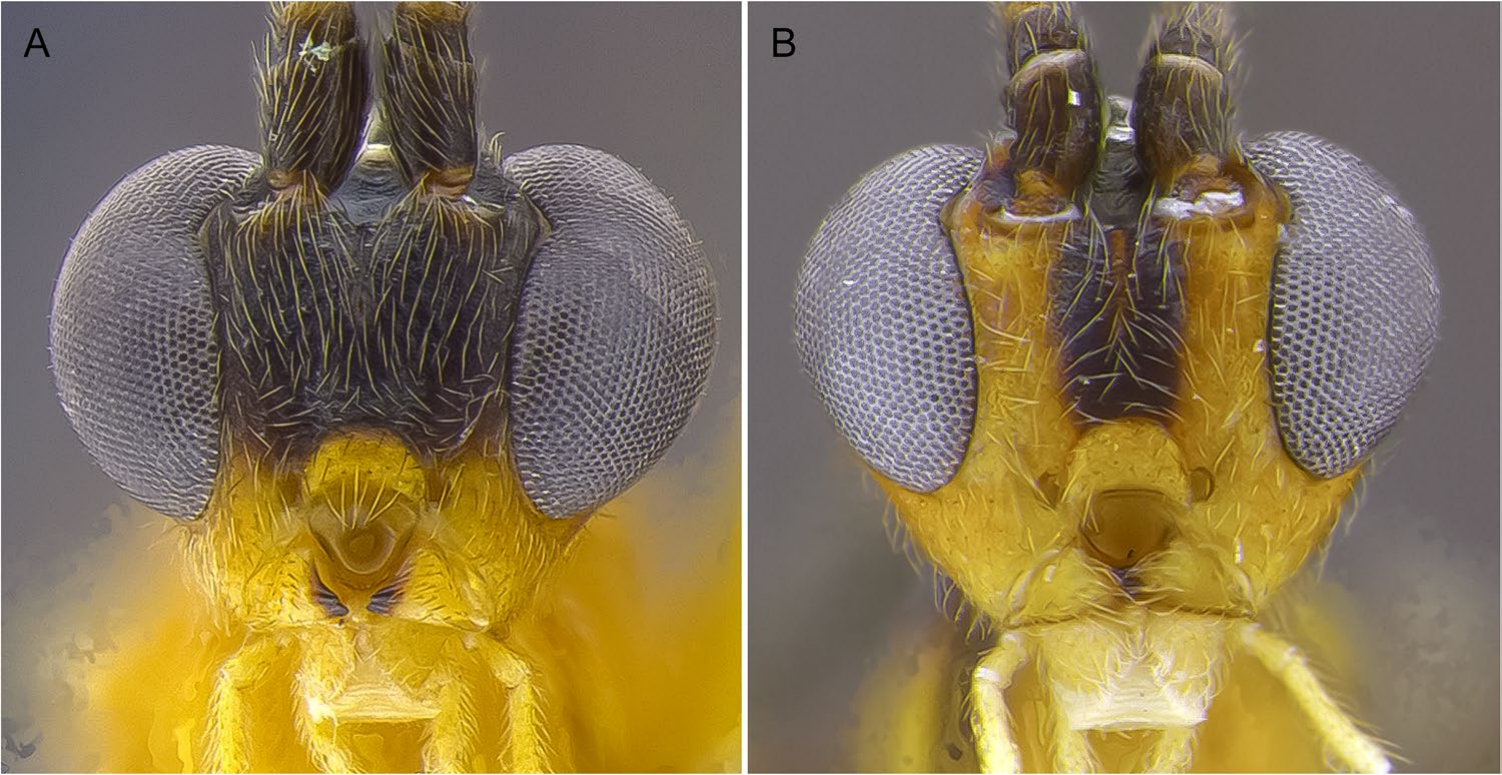
Face. **A** *Aleiodes aclydis*; **B** *Aleiodes speciosus*

**Fig. 13:**
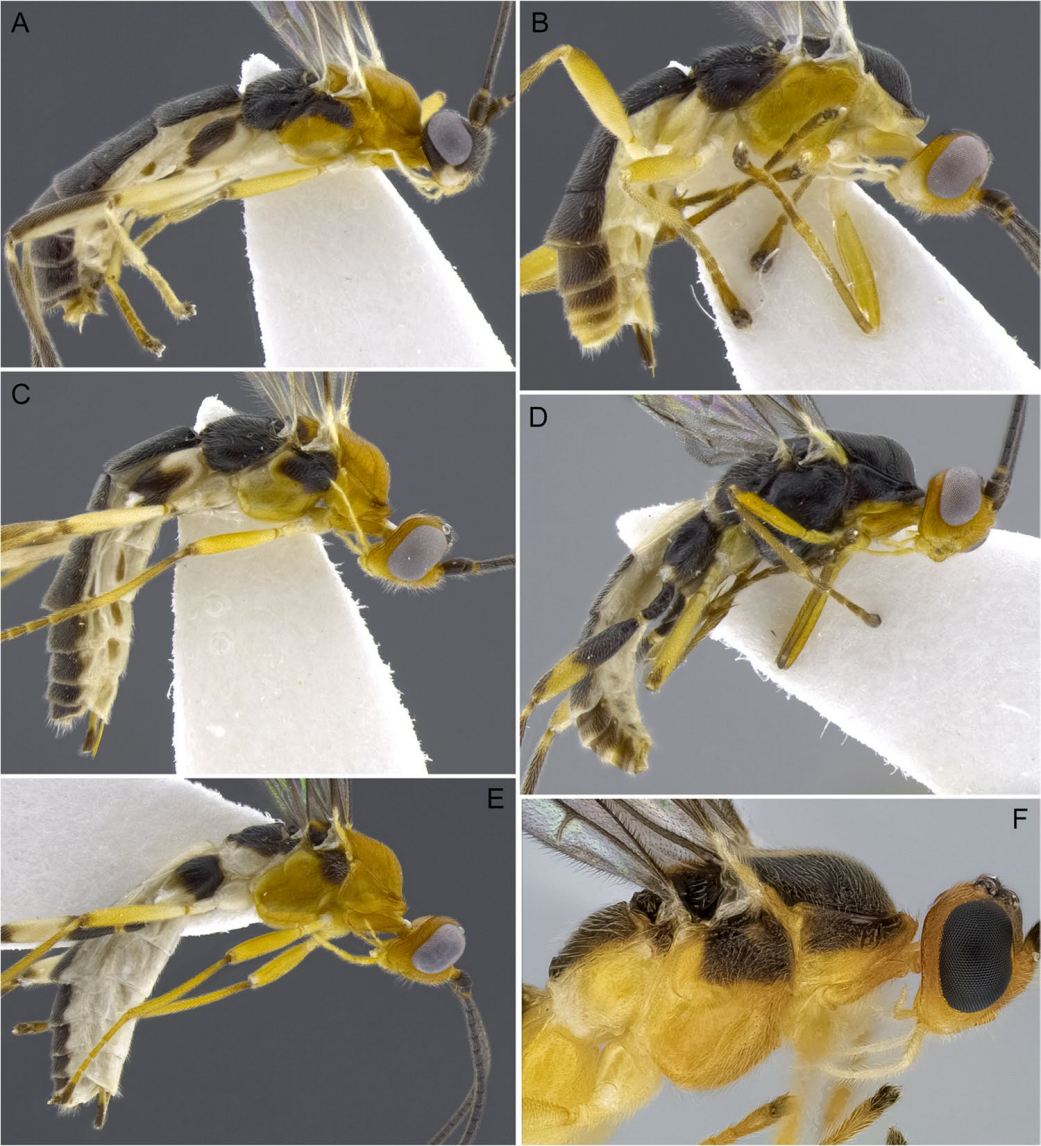
Habitus, lateral. **A** *Aleiodes albigena*; **B** *Aleiodes shakirae*; **C** *Aleiodes townsendi*; **D** *Aleiodes tzantza*; **E** *Aleiodes kingmani*; **F** *Aleiodes japi*

**Fig. 14:**
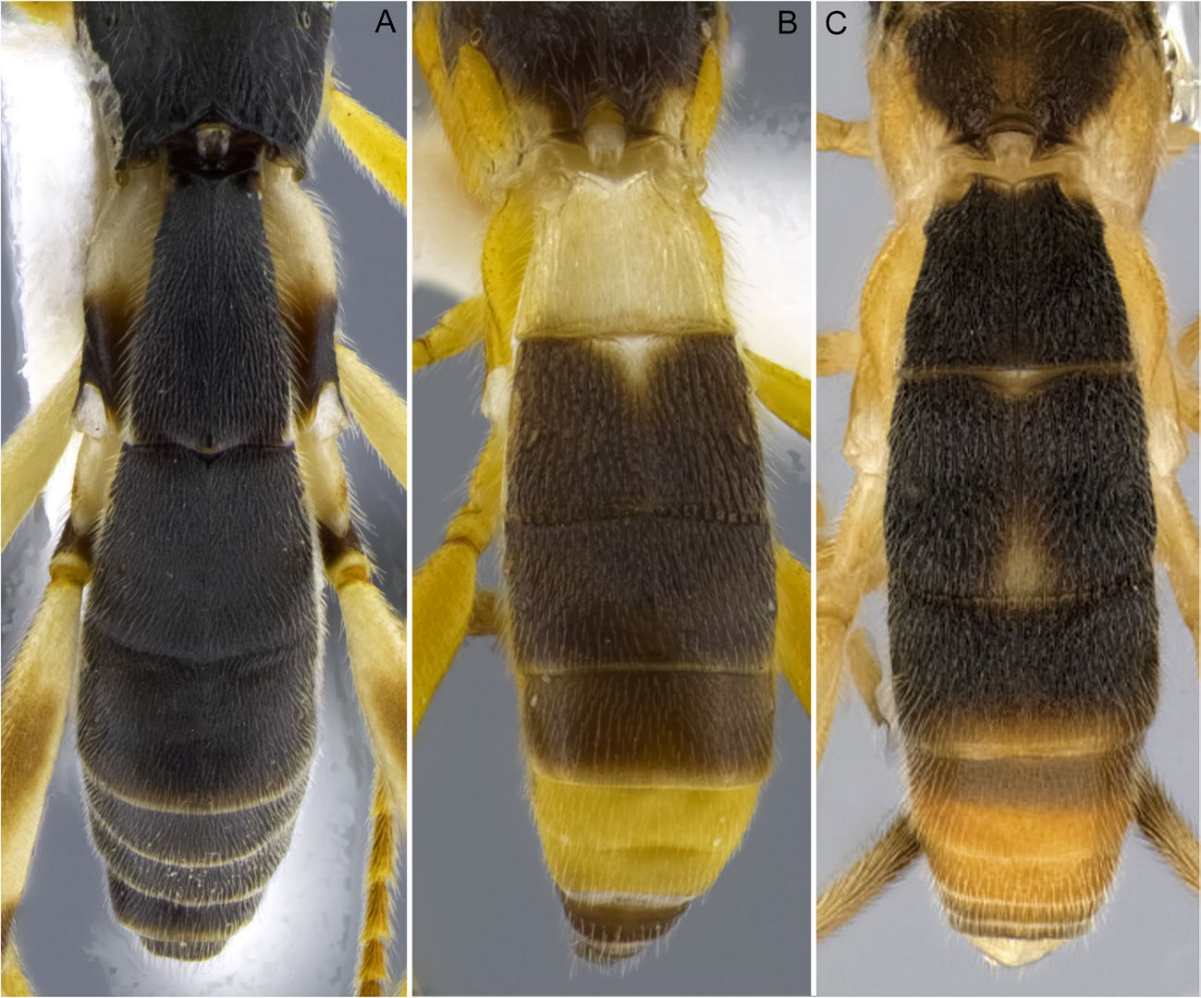
Metasoma, dorsal. **A** *Aleiodes shakirae*; **B** *Aleiodes speciosus*; **C** *Aleiodes japi*

**Fig. 15:**
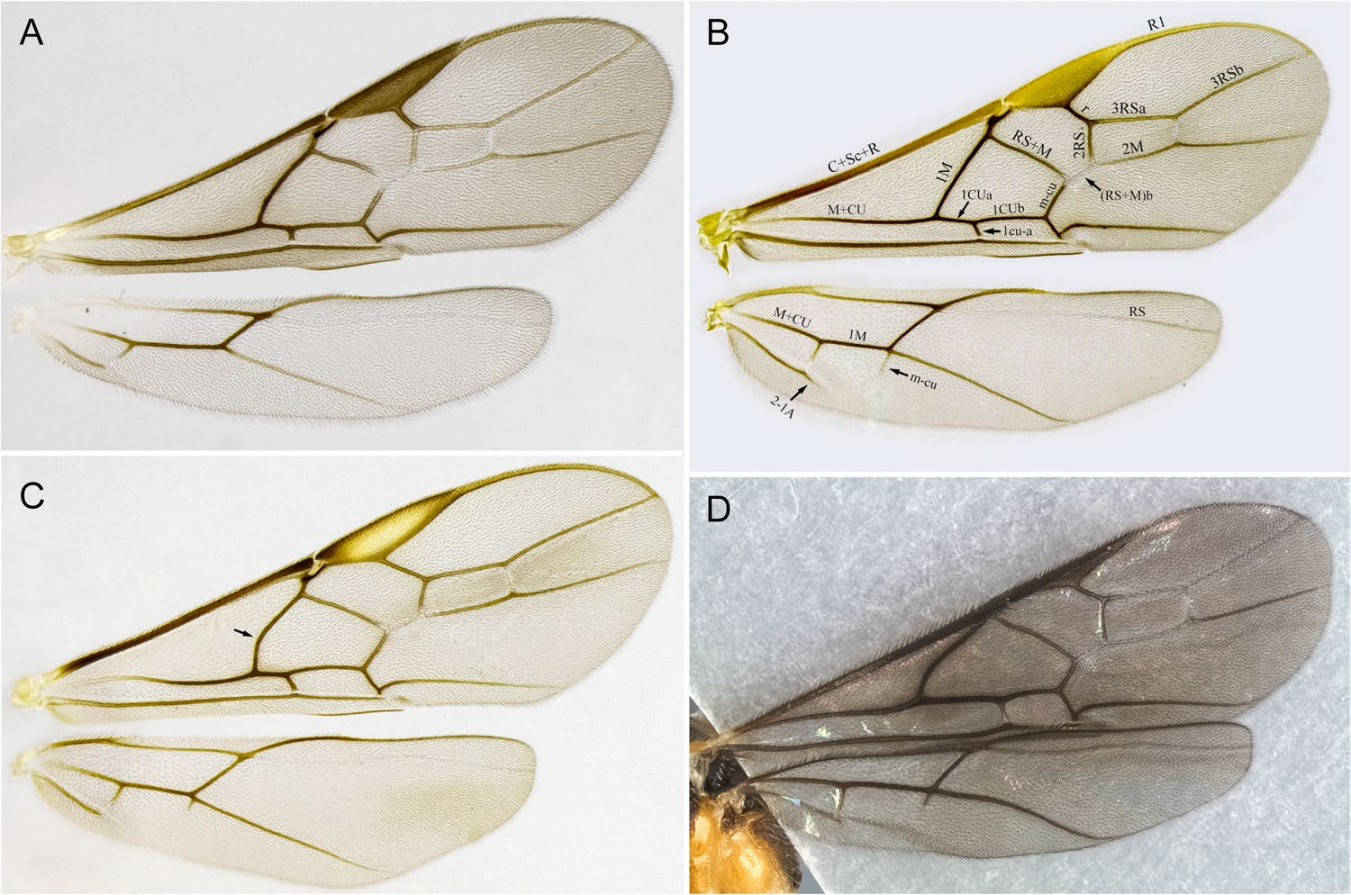
Wings. **A** *Aleiodes atripileatus*; **B** *Aleiodes luteosicarius*; **C** *Aleiodes shakirae*; **D** *Aleiodes japi*

**Fig. 16:**
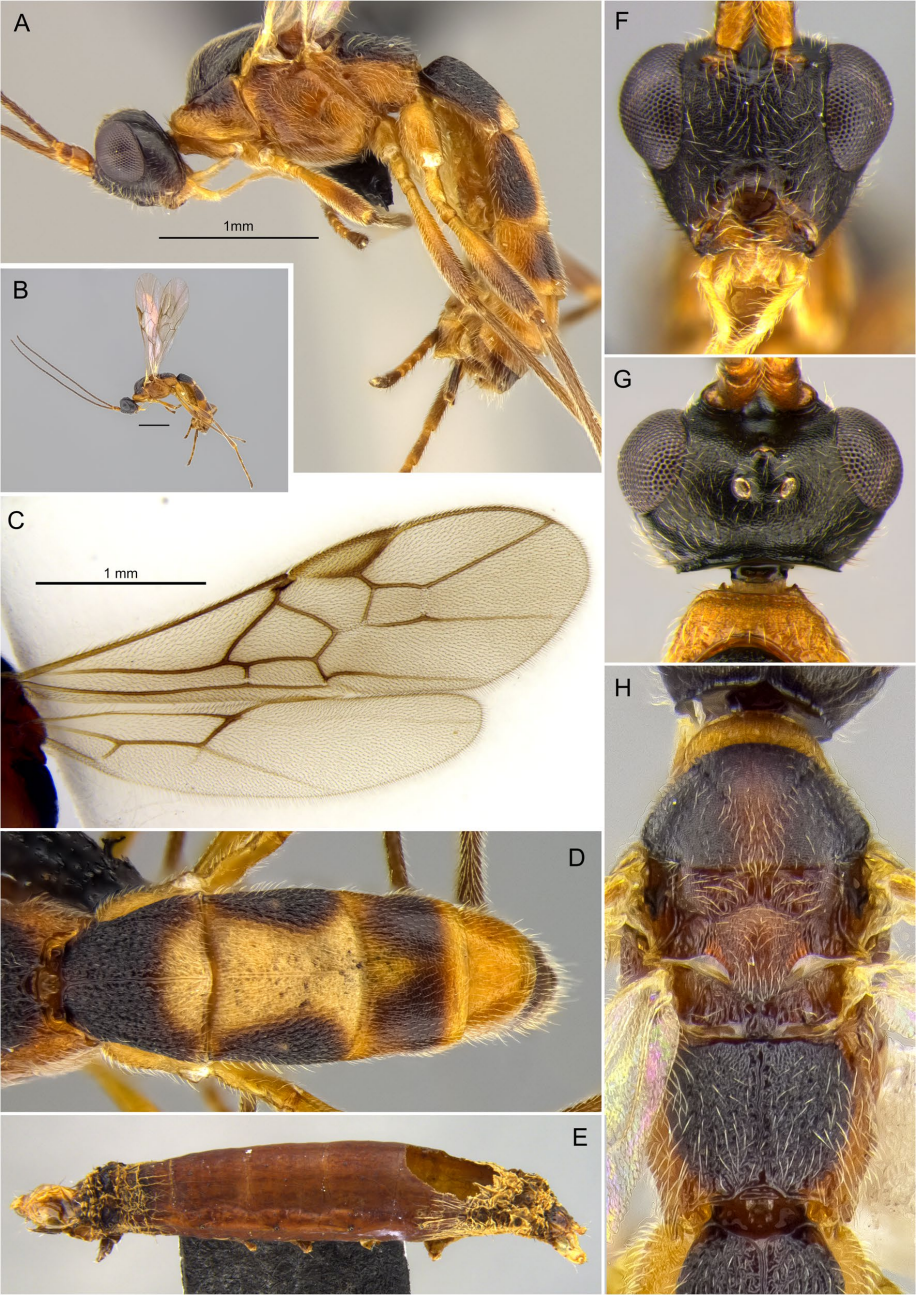
*Aleiodes ceres* **sp.n**. (A, B) Habitus, lateral; (C) wings; (D) metasoma, dorsal; (E) mummified remains of the host, *Spodoptera eridania*; (F) face; (G) head, dorsal; (H) mesosoma, dorsal

**Fig. 17:**
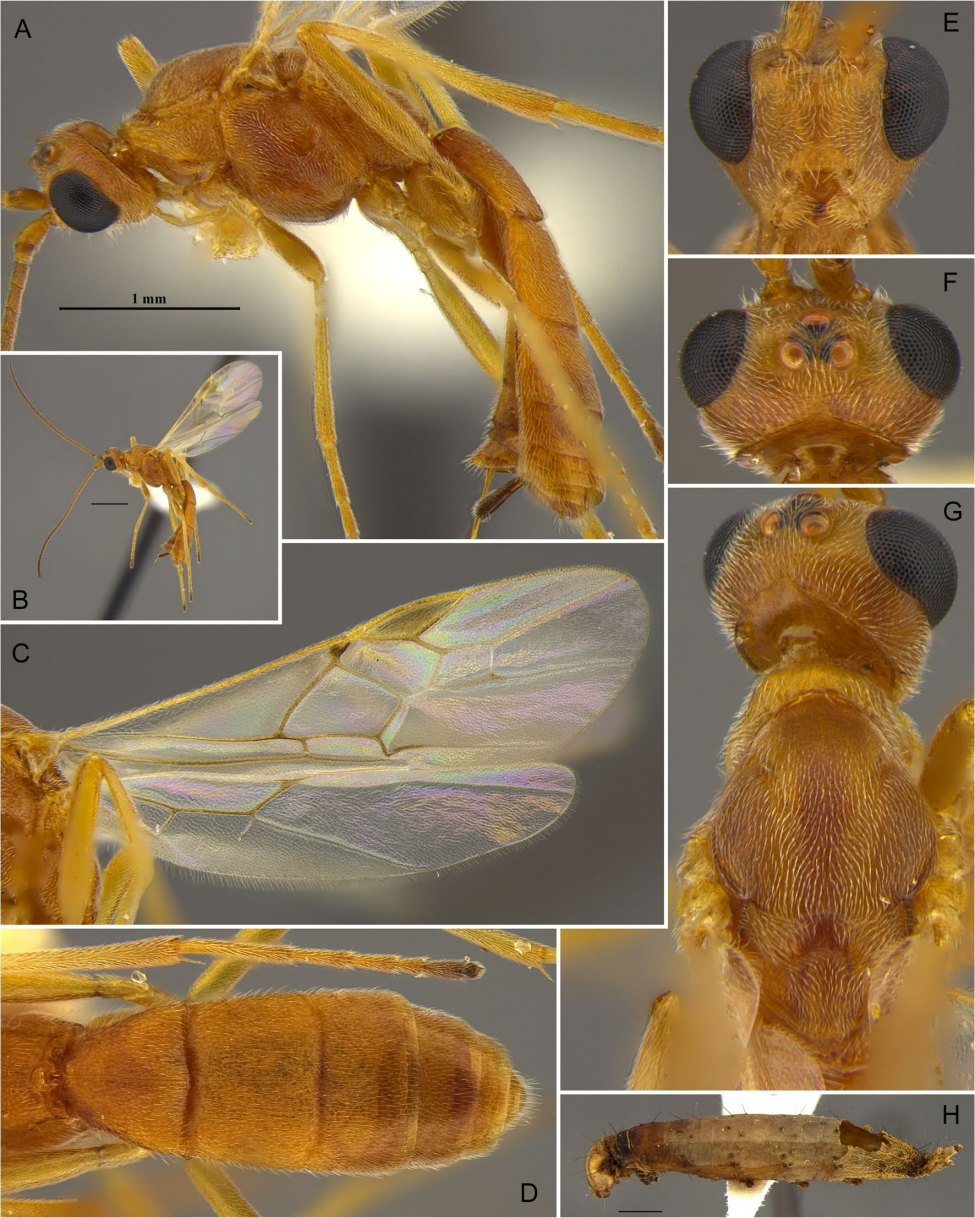
*Aleiodes laphygmae* (Viereck, 1912)*.* (A, B) Habitus, lateral; (C) wings; (D) metasoma, dorsal; (E) face; (F) head, dorsal; (G) mesosoma, dorsal; (H) mummified remains of the host, *Spodoptera frugiperda*

- 1.  Ocelli small, ocelli–ocular distance longer than width of lateral ocellus (Fig 2A, B) … 2
- Ocelli moderate-sized, ocelli–ocular distance equal to or shorter than width of lateral ocellus (Fig 2C, D) … 14
- 2(1). First and/or second metasomal terga with median carina present (Fig 3A); ovipositor sheaths at most 2/3 length of hind basitarsus … 3
– First and second metasomal terga with median carina absent (Fig 3B); ovipositor about 2× length of hind basitarsus; Geometridae hosts … ***albiterminus*** Townsend
- 3(2). Malar space about as long as width of mandible base (Fig. 4A); head mostly black to dark brown, except for a crescent moon-shaped brown mark vertex, contrasting to thorax mostly yellow; Geometridae hosts … ***arbitrium*** Townsend
– Malar space at least 1.25× width of mandible base (Fig. 4B); head and thorax coloration not as above … 4
- 4(3). Occipital carina weak and interrupted mid-dorsally (Fig 9A, C) … 5
– Occipital carina complete and well-defined at mid-dorsally (Fig. 10B, D) … 10
- 5(4). Mesopleuron with central disc lacking setae, smooth and shining (Fig. 5A, B) … 6
– Mesopleuron with central disc mostly setose and granulate (Fig. 5C, D) … 8
- 6(5). Tergite 2 mostly black with white markings, hind coxa black (Fig. 6A); hind wing vein M+CU shorter than 1M (as in Fig. 15C)… 7
– Tergite 2 entirely whitish yellow, hind coxa yellowish (Fig. 6B); hind wing vein M+CU about as long as 1M (as in Fig. 15A); Geometridae hosts… ***yanayacu*** Shimbori & Shaw
- 7(6). Head, pronotum, propleuron, and scutellum orangish yellow (Fig. 7A), except ocellar triangle black; tergite 1 entirely white; tergite 2 with median carina complete; Geometridae hosts … ***mirandae*** Shimbori & Shaw
– Head and thorax black, except reddish brown mark on temples, just behind eyes; tergite 1 white with large black medial spot (Fig. 7B); tergite 2 with median carina incomplete, not reaching the end of tergum; Noctuidae hosts … ***napo*** Shimbori & Shaw
- 8(5). Entirely honey yellow except ocellar triangle dark brown (Fig. 8A); host unknown … ***argentiniensis*** Shimbori & Martinez
– Body with extensive black markings on mesosoma and metasoma (Fig. 8B) … 9
- 9(8). Head orange, except for black ocellar triangle, contrasting with mostly black body (Fig. 9C, D); mesopleuron entirely black (Fig. 9E); ovipositor sheaths shorter than 1/2 length of hind basitarsus; hind wing vein m-cu distinct (as in Fig. 15D); Zygaenidae hosts … ***onyx*** Shimbori & Shaw
– Head mostly yellowish brown with large black semicircular spot on occiput, vertex and ocellar triangle also black (Fig. 9A); mesopleuron with ventral 1/2 yellowish brown, dorsally black (Fig. 9B); hind wing vein m-cu absent (Fig. 15A) or at most weakly indicated by infumate pigmentation; Noctuidae hosts … ***atripileatus*** Townsend
- 10(4). Pronotal collar yellowish brown or honey yellow (Figs. 16(G), 17(G)) … 11
– P ronotal collar black (Fig. 10B, D) … 13
- 11(10). 39–46 antennomeres; hind coxa rugose dorsally … ***bimaculatus*** Shimbori & Shaw
– 31–36-antennomeres; hind coxa granulate dorsally … 12
- 12(11). Ocelli small, ocell-ocular distance about 2× longer than diameter of lateral ocellus (Fig. 16(G)); stigma pale brown, lighter centrally (Fig. 16(C)); head usually dark brown or black, metasomal terga 1–3 frequently with dark brown areas (Fig. 16(A, D)); Noctuidae hosts… ***ceres*** sp. n.
– Ocelli larger, ocell-ocular distance about as long as diameter of lateral ocellus (Fig. 17(F)); stigma brown with yellow spots at base and apex (Fig. 17(C)); body color entirely honey yellow (Fig. 17(A, D, E, G)); Noctuidae hosts … ***laphygmae*** (Viereck)
- 13(10). Mesoscutum with square orangish brown mark postero-medially (Fig. 10D); head mostly dark brown to black with crescent moon-shaped honey brown area bordering eyes at temples (Fig. 10C, D); Geometridae hosts … ***nubicola*** Shimbori & Shaw
– Mesoscutum entirely black (Fig. 10B); head color variable, mostly yellowish with black occiput and vertex, and dark brown frons and face medially (Fig. 10A, B); Geometridae hosts … ***cacuangoi*** Shimbori & Shaw
- 14(1). Mesosoma and metasoma mostly honey yellow (Fig. 11A–C), sometimes with dark marks on mesoscutum … 15
– Propodeum and most of metasomal terga black or dark brown (Fig. 13A, B, C), or sometimes first tergite white (Fig. 13D, E); hind coxa sometimes bicolored black and white (Fig. 13A, C, E) … 18 (*A. shakirae* species-group)
- 15(14). Fore wing vein at least 1CUa 2.0× vein 1cu-a; antenna with 43–51 flagellomeres …16
– Fore wing vein 1CUa 1.5× vein 1cu-a; antenna with 36–39 flagellomeres; Erebidae hosts … ***gossypii*** Muesebeck^2^
- 16(15). Hind wing vein m-cu present as pigmented not tubular vein; fore wing vein 1CUa 1.8× 1CUb, vein r 0.5–0.7× vein 2RS; T1 and T2 with complete median carina … 17
– Hind wing vein m-cu absent; fore wing vein 1CUa 1.3× vein 1CUb, vein r 0.9× vein 2RS; T1 and T2 with median carina weak or absent apically; a gregarious parasitoid of Erebidae… ***leptocarina*** Fortier
- 17(16). Hind wing vein 2-1A absent; diameter of lateral ocellus roughly as long as ocell–ocular distance (Fig. 11B) … ***falloni*** Shimbori & Shaw
– Hind wing vein 2-1A present (Fig. 15B); diameter of lateral ocellus about 3× ocell–ocular distance (Fig. 11D); Erebidae hosts … ***luteosicarius*** Shimbori & Shaw
- 18(14). Malar space short, length 0.7× width of mandibular base (Fig. 12A); median carina absent on propodeum; Geometridae hosts … ***aclydis*** Townsend
– Malar space moderately wide, at least slightly longer than width of mandibular base (Fig. 12B); median carina present on propodeum … 19
- 19(18). Mesoscutum and scutellum honey yellow, hind coxa bicolored black and white (Fig. 13A, C, E) … 20
– Mesoscutum and scutellum partially to mostly black, hind coxa one color, either black (Fig. 13D) or whitish-yellow (Fig. 13B, F) … 22
- 20(19). Head honey yellow, ocellar triangle dark brown (Fig. 13C, E); fore wing vein 1M strongly curved basally (Fig. 15C) … 21
– Head dark brown, gena white (Fig. 13A); fore wing vein 1M almost straight or weakly and evenly curved (as in Fig. 15A); Erebidae hosts … ***albigena*** Shimbori & Shaw
- 21(20). First metasomal tergite about 2× longer than its apical width, dark brown to black (Fig. 14A); hind coxa basally white and apically black (Figs. 13C, 14A); Geometeridae hosts … ***shakirae*** Shimbori & Shaw
– First metasomal tergite about as long as apical width, white with small black spot mid-apically; hind coxa black basally and apically white (Fig. 13E); Geometridae hosts … ***townsendi*** Shimbori & Shaw
- 22(19). First metasomal tergite dark brown, not contrasting with reminder terga (14C) … 23
– First metasomal tergite white, contrasting with remainder mostly dark brown metasoma (Figs. 13D, 14B) … 24
- 23(22). Fore wing vein 1CUa about 0.45× as long as vein 1CUb (as in Fig. 15A); Noctuidae hosts… ***tzantza*** Shimbori & Shaw
– Fore wing vein 1CUa about 1.3× as long as vein 1CUb (Fig. 15D); Geometridae hosts …***japi*** Shimbori & Penteado-Dias
- 24(22). Mesopleuron, metapleuron, hind coxa, and propodeum medially and anteriorly smooth; mesopleuron except for anterior corner and hind coxa orangish; Geometridae hosts … ***speciosus*** Townsend
– Mesopleuron, metapleuron, hind coxa and propodeum granulate; mesopleuron and hind coxa black (Fig. 13D); Geometridae hosts … ***kingmani*** Shimbori & Shaw

**Comments on biology of Neotropical species**. Information regarding association with hosts is quite abundant. Of the 36 species, 24 have host records (Table 1). The main hosts for *gastritor* are caterpillars in the families Geometridae, Noctuidae, and Erebidae, with Zygaenidae, Uraniidae and Crambidae recorded as hosts for one species each. The *shakirae* species-group attacks exclusively geometrids (Table 1). Zaldívar-Riverón et al. (2008) proposed that Geometridae host is a basal condition in the subgenus *Aleiodes*, which is congruent with the basal position of the *shakirae* species-group*.*

### Taxonomic treatments

***Aleiodes ceres*** **Shimbori sp. n.**

http://zoobank.org/707EBA08-7291-4C29-A0EB-3908E61959D0

Figure 16(A–H).

#### Material examined

Holotype, female. “Brazil, Paraná, São José dos Pinhais. 25°36′49″S 49°08′01″W. 01.II.2018. Emerged from *Spodoptera eridania* on soybean leaves. ES.31B” (DCBU 518.066).

Paratypes: 2 females and 1 male, same data as holotype (DCBU 518.067–518.069); 4 females and 4 males, same data as holotype except “1.I.2018…” (DCBU 518.070–518.077); 2 females and 1 male, same data as holotype except “25°36′44.68″ S 49°08′17.31″ W. 1–15.II.2017. Soja trans (bt-rr2)…” (DCBU 518.078–518.080).

#### Description

Female. Body length 3.9–4.5 mm. Fore wing length 3.2–3.7 mm.

Head (Fig. 16(A, F, G)). In dorsal view: eye length/temple 1.35–1.52; eye height/head width 0.37–0.41; eye height/minimum distance between eyes 0.68–0.75; OD/POL 0.71–0.86; OOL/OD 2.16–2.56. Frons not excavated and without lateral carina. Occipital carina complete, not interrupted dorsally and ventrally touching hypostomal carina. Occiput in dorsal view nearly straight, not indented medially. Mid-longitudinal crest at upper face absent. In frontal view: hypoclypeal depression/face width 0.34–0.41; malar space/eye height 0.55–0.64; malar space/mandibular base width 1.2–1.4; face height/width 0.54–0.66; clypeus height/width 1.8–2.0. Clypeus convex, coriaceous. Sculpture of head shiny finely granular-coriaceous.

Antenna. Antennal segments 31–33. Antenna/body length 0.91–1.07. Scape/pedicel length 1.78–1.88. Length of first/second flagellomere 0.84–0.92. Fourth flagellomere length/apical width 2.6–3.3. Tip of apical segment of antenna lanceolate.

Mesosoma. Length/height 1.69–1.77. Width of mesoscutum/width of head 0.81–0.86. Pronotal collar short, nearly as long as vertex. Prescutellar sulcus with complete median carina plus 3 pairs of lateral carinae. Mesoscutum posterior border with incomplete carina. Metanotum with small mid-pit posteriorly, delimited by carinae. Mid-longitudinal carina of propodeum complete. Ventral mid-line of mesopleuron set within very shallow smooth sulcus; pit at ventral mid-line present posteriorly. Notauli distinct anteriorly, finely crenulate; posteriorly indistinct. Sternaulus weakly indicated. Sculpture of mesosoma mostly granulate. Pronotum rugose laterally. Mesopleuron with small polished spot mid-dorsally. Mesoscutellar trough entirely costate. Metanotum mostly smooth and weakly crenulate. Propodeum mostly granulate-rugose.

Wings. Membrane uniformly and densely setose. Fore wing: Stigma length/height 3.77–3.85. Vein r/2RS 0.51–0.62. Vein r/RS + Mb 0.63–0.77. Vein 3RSa/2RS 1.33–1.57. Vein 3RSa/2 M 0.74–0.84. Vein 3RSa/3RSb 0.34–0.42. Vein 1CUb/1CUa 2.9–3.4. Vein 1CUa/1cu-a 1.07–1.15. Vein 1 M weakly and evenly curved. Vein RS + Ma weakly sinuate. Vein M + CU slightly curved at apical half. Vein 1-1A only slightly curved basally. Vein 1a absent. Second submarginal cell trapezoidal. Hind wing: Vein RS running nearly parallel to wing margin, weakly sinuate mid-basally. Marginal cell narrowest at basal 1/3. Vein M + CU/1 M 1.10–1.17. Vein 1 M/r-m 2.01–2.29. Vein m-cu present, spectral. Vein m-cu position relative to vein r-m interstitial. Vein 2-1A absent. Basal cell evenly setose with a small bare spot posteriorly.

Hind legs. Femur length/width 6.1–6.4. Length of tibia/tarsi 2.35–2.55. Length of basitarsus/tarsomere 2 1.9–2.0. Length of basitarsus/inner tibial spur 3.6–5.2. Sculpture of hind coxa dorsally granulate. Tarsal claws finely pectinate basally, with wide gap until base of claw.

Metasoma. T1 length/apical width 1.0–1.1; apical/basal width 2.25–2.46. Mid-longitudinal carina extending until basal 0.2 of T3. Metasoma sculpture T1 and T2 striate-rugose, remainder terga granular-coriaceous. Ovipositor sheath/hind basitarsus 0.4–0.5. Apex of ovipositor sheaths truncate.

Color (Holotype—female). Head dark brown, except palpi light pale yellow and mandibles pale brown. Antenna with flagellum brown; scape and pedicel mostly honey yellow, light brown dorsally. Mesosoma mostly honey yellow; mesoscutum and propodeum dark brown; metanotum and scutellum light brown, darker medially. Metasoma light pale yellow ventrally; T1–2 dark brown with large pale yellow mark medially, starting at posterior 1/4 of T1 and extending posteriorly to cover most of T2 medially, and reaching anterior 1/2 of T3 as a small triangular spot; T3 mostly brown to dark brown with honey yellow borders, remainder terga mostly honey yellow with brown spots on T5–7 medially. Wings faintly tinged brown; veins mostly brown; stigma pale yellow with infuscate borders. Legs honey yellow basally (Coxae, trochanter, trochantellus, and basal half of femur), apical half of femur, tibia and 5th tarsomere brown, tarsomeres 1–4 light brown. Ovipositor sheaths dark brown.

Male. Body length 3.7–4.5; fore wing length 2.9–3.7. Similar to females in most aspects, except for the antenna which is longer than body (about 1.2 ×), compared with the antenna about as long as body in females. Eyes and ocelli relatively larger in males, OOL/OD 1.92–2.12 and eye length/temple 1.46–1.67.

**Biology**. Specimens of *A. ceres* collected in 2019/2020 soybean crop season have been kept in the laboratory for over 64 generations without losing their biological traits. In the laboratory, *A. ceres* can develop in the hosts: *S. cosmioides*, *S. eridania*, and *S. frugiperda* with their hosts being reared on a natural or artificial diet. Females of *A. ceres* can parasitize caterpillars from the first to third instar; however, better parasitism and emergence rates were observed in caterpillars of the first and second larval instar. When offered the second instar of *S. eridania*, *A. ceres* parasitized on average 9.07 ± 0.78 caterpillars in 24 h. In 3 days, a total of 26.0 ± 1.47 caterpillars were parasitized. The development time (egg-adult) was 16.58 ± 0.09 days and the emergence rate was 87.44 ± 2.59 at 25 ± 2 °C, 70 ± 10% RH.

This species seems to be closely associated with caterpillars of the *Spodoptera* complex because no parasitism was recorded in other species like *Anticarsia gemmatalis* Hübner, *Chrysodeixis includens* (Walker), *Ephestia kuehniella* (Zeller), *Helicoverpa armigera* (Hübner), and *Mythimna sequax* (Franclemont).

**Etymology**. The choice for the name of the new species was made in a public contest, with the objective of raising awareness and interest of the general public, especially middle and high school students, for taxonomic research and its relevance to biological control. The contest was held during a community outreach fair “ESALQSHOW,” on the 6th and 7th of October, 2022, on the campus of the “Luiz de Queiroz College of Agriculture (ESALQ),” of the University of São Paulo. For all participants, a brief explanation of the rules of zoological nomenclature was given, in addition to the economic and ecologic importance of the new species and the consequences of the gap in taxonomic knowledge. The name chosen by one of the public contest participants was given in reference to the goddess of agriculture, Ceres, of Roman mythology.

**Diagnosis**. Very small ocelli, ocell-ocular distance more than 2 × longer than diameter of lateral ocellus in females (about 2 × in males); body color variable, mostly honey yellow with dark brown marking at mesoscutum, propodeum and metasoma, pronotum and mesopleuron always honey yellow, head nearly always black; stigma entirely pale brown; longitudinal carina at metasomal tergum 3 absent or shortly indicated anteriorly, complete at terga 1 and 2.

**Comments**. The new species differs from *A. laphygmae* in having much smaller ocelli. The ocell-ocular distance is more than two times longer than the diameter of the lateral ocellus (in contrast to an ocell-ocular distance equal to or slightly shorter than the diameter of the lateral ocellus in *A. laphygmae*). The metasomal tergum 3 has a nearly complete longitudinal carina in *A. laphygmae*, but in the new species, the longitudinal carina is absent or only weakly indicated anteriorly. The new species also has a much more extensive darker coloration, including head (females), mesonotum, propodeum, and metasomal terga 1–4, compared with the entire honey-yellow body of *A. laphygmae*. The new species also resembles *Aleiodes nubicola* Shimbori and Shaw 2014, in having a dark head with small ocelli, and complete occipital carina. The two species differ in the coloration of pronotum, which is entirely dark brown in *A. nubicola*, as compared to entirely yellow in *A.* ceres **sp.n.** A species of Geometridae is the only known host for *A. nubicola,* while *A. ceres* **sp.n.** is known to attack *Spodoptera eridania* (Noctuidae), which also results in very different caterpillar mummies produced by each parasitoid species (see Fig. 91 in Shimbori and Shaw 2014 for comparison).

The recent record of *A. laphygmae* from Argentina (Valverde et al. 2012) has to be revisited in view of our findings. Considering geographic distribution, host and plant food associations, and morphological features, the identity of the specimens recorded from there are likely to be *Aleiodes ceres* **sp.n.** Sequences of *Aleiodes ceres* **sp.n.** from Argentina included in the phylogeny were mined from BOLD, and there is no evident connection with specimens reported by Valverde et al. (2012). The images of one of the specimens corroborates identification as *A. ceres* **sp.n.**

**Distribution**. Argentina^3^ and Brazil.

Records from Argentina are based on DNA Barcode sequences deposited in public databases (BOLD record IDs: GMAGR178-15; GMARM1238-14; GMAGP1961-15; GMAGR182-15). The similarity between sequences from Argentinian and Brazilian specimens ranges from 98.3 to 99.1%. Our ML phylogeny also supports the conspecificity of Argentinian and Brazilian specimens. All Argentinian specimens are deposited at Museo Argentino de Ciencias Naturales Bernardino Rivadavia, in Buenos Aires, Argentina, and are assigned a single BIN (Barcode Index Number), BOLD:ACN2401 (available at: http://boldsystems.org/index.php/Public_BarcodeCluster?clusteruri=BOLD:ACN2401).

#### DNA barcode of the holotype and two paratypes

AATTTTATATTTTATTTTTGGAATATGAGCAGGAATAATTGGAATATCAATAAGTTTAATTATTC GATTAGAATTAAGAACAGGAGGAAGAATTTTAAAAAATGACCAAATTTATAATGGAATAGTA ACTTTACATGCTTTTATTATAATTTTTTTTATAGTAATACCAATTATAATTGGGGGTTTTGGAAA TTGATTAATTCCTTTAATGTTAGGAGCCCCTGATATAGCTTTCCCACGAATAAATAATATAAGA TTCTGATTATTAATCCCTTCTTTAATACTTTTATTAATTAGAGGATTAATCAATACAGGAGTAGG GACTGGTTGAACAATATACCCTCCATTATCATCATTAATTGGTCATAACGGAATTTCTGTAGAT ATATCAATTTTTTCCTTACATCTGGCGGGAGCTTCTTCAATTATAGGAGCAATTAATTTTATTTC AACAATTTTCAATATAAATTTAATAAAAATTAAAATAGATCAAATTATATTATTAATTTGATCTA TTTTAATTACTACAATTTTATTACTTTTATCTTTACCAGTCCTGGCTGGCGCTATTACTATACTAC TAACAGACCGAAATTTAAATACAGCATTTTTTGACTTTTCAGGGGGAGGAGACCCCATTTTATT CCAACATCTTTTC.

***Aleiodes laphygmae*** **(Viereck, 1912)**

Figure 17(A–H).

*Rogas laphygmae* Viereck 1912, 43: 581.

#### Diagnosis

Ocelli moderate-sized, ocell-ocular distance about as long as the diameter of lateral ocellus; body color entirely honey yellow; stigma brown with yellow spots at base and apex; longitudinal carina at metasomal tergum 3 present and extending nearly over the entire length of tergite; body length 4.0–5.0 mm; antenna with 33–35 antennomeres; the angle between fore wing vein r and basal ventral line of stigma about 160 degrees (Fig. 17), both appearing nearly in the same line.

**Biology**. The species is a parasitoid of the early stages of a few caterpillar species in the family Noctuidae, namely *Mythmna unipuncta* (Haw.), *Spodoptera exigua* (Hbn.), *S. frugiperda* (Smith), and *S. orthogonali* (Guen.).

**Distribution**. Southern USA, Cuba, and Nicaragua. Recent records from Argentina are most likely of *Aleiodes ceres* **sp.n.**

TAXONOMIC NOTES

***Aleiodes leptocarina*** Fortier 2000

*Aleiodes normwoodleyi* Sharkey, 2021 NEW SYNONYMY

During this study, we established that *Aleiodes normwoodleyi* Sharkey, 2021 is a junior synonym of the previously described species *Aleiodes leptocarina* Fortier 2000. Fortier (2000) described the new species *Aleiodes leptocarina* from Costa Rica, which was notable (at that time) in being the only known *Aleiodes* from Costa Rica to develop gregariously inside large caterpillars. The identity of the host caterpillar was not known at that time, but Fortier (2000) did include a black and white photograph of the large, densely setose host mummy. More recently, Sharkey (2021) in Sharkey et al. (2021) described a similar “new species,” which also exhibited gregarious development (80 specimens emerged from one large caterpillar mummy of *Dysschema vidua* (Erebidae)). Sharkey’s “minimalist” species description was based on a DNA sequence, one photograph, the host association with *Dysschema vidua*, and only one stated morphological difference: the antennal flagellum was stated to be “melanic” (black) in *A. normwoodleyi*, as opposed to being “honey yellow” in *A. leptocarina*. During this study, we re-examined the holotype and paratype series for *A. leptocarina* Fortier, and observed that the flagellum is not yellow but instead is entirely black in all the type specimens. We believe that confusion was created by an error in Fortier’s (2000) description of *A. leptocarina*, where the flagellum color was incorrectly listed as being “honey yellow.” The images in Fortier’s paper were all done by scanning electron microscopy, so the resulting photos are in gray tones and it is impossible to tell the antenna color from the images. While the caterpillar host for *A. leptocarina* was never determined, its densely hairy appearance is consistent with it possibly being a caterpillar of *Dysschema vidua*. Additionally, one of us (SRS) has previously identified in 2005 a long series of gregarious specimens reared from a mummified caterpillar of *Dysschema vidua* as being *A. leptocarina* (by direct comparison with the type series). This host association was reported to Dan Janzen and was posted on the *Caterpillars of the ACG* website for many years (until the species association was later changed to *A. normwoodleyi*), presumably on the basis of the wasps having a black flagellum (and the erroneous assumption that *A. leptocarina* specimens have a yellow flagellum). Therefore, we regard *Dysschema vidua* to be a previously known host record for *A. leptocarina* (in fact, the only known host for it so far established). We regard the species concept of *A. normwoodleyi* to be based on an (understandable) misidentification; therefore, the molecular sequence presented by Sharkey (2021) is presumed to actually be the barcode sequence for *A. leptocarina* Fortier. No other barcode data for *A. leptocarina* were found on the BOLD website for comparison.

### Supplementary Information

Below is the link to the electronic supplementary material.

Supplementary file1 (PDF 518 KB)

## Data Availability

All data generated or analysed during this study are included in this published article or available in public databases.

## Footnotes

1 Species named in Sharkey et al. (2021) are not considered for any morphological discussion as they lack morphological descriptions, except for one image of the adult insect in lateral view.

2 *Aleiodes bonariensis* should key here. However, the condition of the holotype is poor, with head and hind wings missing (Shimbori and Martínez 2016), preventing inclusion in the identification key.

3 Based on DNA barcode sequences in public databases with the following access numbers: GMARM1238-14; GMAGR178-15.
