# Supplementary material for: A New Species of Aleiodes Wesmael (Braconidae, Rogadinae) with Potential for Biological Control of Spodoptera spp. (Lepidoptera, Noctuidae), and Notes on the Definition of the gastritor, circumscriptus, and Related Species-Groups
Source: Neotrop Entomol. 2023 Sep 19;52(6):1064–87. doi: 10.1007/s13744-023-01076-8 (PMC10695885; doi:10.1007/s13744-023-01076-8)
Supplement: Supplementary file 1 — Supplementary file1 (PDF 518 KB) [file 13744_2023_1076_MOESM1_ESM.pdf]

Article title: A new species of *Aleiodes* Wesmael (Braconidae, Rogadinae) with potential for biological control of *Spodoptera* spp. (Lepidoptera, Noctuidae), and notes on the definition of the *gastritor*, *circumscriptus* and related species-groups.

Journal: Neotropical Entomology

Authors: Eduardo Mitio Shimbori*, Tamara Akemi Takahashi, Isabela Midori Watanabe, Angélica Maria Penteado-Dias, Luís Amilton Foerster, Scott Richard Shaw, José Roberto Postali Parra

*Departamento de Entomologia e Acarologia, Escola Superior de Agricultura "Luiz de Queiroz" (ESALQ), Universidade de São Paulo (USP), Piracicaba, São Paulo, Brazil; Colección Nacional de Insectos, Instituto de Biología, Universidad Nacional Autónoma de México (UNAM). Email: shimbori@gmail.com

Appendix 1. Specimens included in the phylogenetic analyses with respective BOLD ID and/or GenBank accession number, and corresponding BIN (Barcode Index Number)

Species	Country	BOLD	GenBank	BIN
Aleiodes abraxanae	UK	ASQSP978-10	HQ551264	AAL6366
Aleiodes achingae	Thailand	ASQSP235-08	JF963431	AAG7597
Aleiodes adorabelleae	Thailand	ASQSP222-08	JF963436	AAG7589
Aleiodes adrianaradulovciae	Costa Rica	GMCRZ087-14	—	AAG1413
Aleiodes adrianforsythi	Costa Rica	ACGBA7882-17	MH496735	ADJ1086
Aleiodes* af. *seriatus	France	—	MN968696	AAB6027
Aleiodes* af. *seriatus	France	ASQSP551-08	—	AEH9474
Aleiodes* af. *seriatus	Russia	ASQSP096-08	JF962858	AAB6028
Aleiodes agagg	Thailand	ASQSR132-11	JN278318	ABY8066
Aleiodes agnespeelleae	Costa Rica	JICCG245-16	—	AEU7732
Aleiodes alaneaglei	Costa Rica	GMCUU772-14	MH272158	AAM5650
Aleiodes alanflemingi	Costa Rica	GMAAT161-16	—	AAM5670
Aleiodes alanhalevii	Costa Rica	PLBAH015-18	—	AAM5683
Aleiodes albitibia	Canada	BBHYM007-09	HM374657	AEB1707
Aleiodes albitibia	Sweden	GBMIN74552-17	KU682238	AAJ2419
Aleiodes alboluteus	Thailand	ASQSQ412-09	HM435164	ACF3847
Aleiodes alejandromasisi	Costa Rica	GMCRG104-13	MH234955	ACG6844
Aleiodes alessandracallejae	Costa Rica	GMCRX533-13	—	ACJ2417
Aleiodes alexsmithi	Costa Rica	ACGBA8966-18	—	AAV7512
Aleiodes alfonsopescadori	Costa Rica	PLAAA6649-18	—	ACR4858
Aleiodes alisundermieri	Costa Rica	JICCE170-16	MH272330	ADB3278
Aleiodes almasolisae	Costa Rica	GMCDG1019-16	MH272300	ADF6889
Aleiodes alternans	French Guiana	ASQSP918-08	JF903049	AAV7508
Aleiodes alternator	UK	ASQSP981-10	HQ551267	AAA7796
Aleiodes alvarougaldei	Costa Rica	PLAAL012-18	—	ABW3270

Species	Country	BOLD	GenBank	BIN
Aleiodes alvaroumanai	Costa Rica	ASHYC4762-10	HQ548691	AAM1443
Aleiodes angelsolisi	Costa Rica	ASHYD2029-10	HQ549184	AAM1722
Aleiodes angulodeum	Thailand	ASQSP413-08	JQ388343	AAH8674
Aleiodes annhowdenae	Costa Rica	ASHYD2835-14	—	AAM5664
Aleiodes antescutum	Thailand	ASQSR080-11	JN278272	AAH8684
Aleiodes apicalis	Turkey	CGBTA785-09	—	AAE5158
Aleiodes apiconigrus	Thailand	ASQSP455-08	JQ388444	AAG7565
Aleiodes apiculatus	UK	ASQSP003-08	KU682239	AAF1016
Aleiodes arbitrium	Ecuador	ASQSQ733-10	HQ551417	AAM9948
Aleiodes ascrobi	Thailand	ASQSQ012-09	JQ388415	AAG7594
Aleiodes assimilis	Turkey	ASQSP078-08	—	ABZ2378
Aleiodes atuin	Thailand	ASQSQ015-09	JQ388412	AAH8677
Aleiodes bachmaduplus	Thailand	ASQSP453-08	JF962539	AAH8682
Aleiodes bachmatriplus	Thailand	ASQSR168-11	JN278339	AAV7461
Aleiodes barnardae	Uganda	ASQBR348-09	—	AAD3610
Aleiodes basipunctatus	Thailand	ASQSR134-11	JN278320	AAV7482
Aleiodes basistriatus	Thailand	ASQSR048-11	JN278265	AAV7465
Aleiodes basutai	Uganda	ASQBR346-09	EF115442	AAJ2411
Aleiodes bicolor	Turkey	CGBTB541-09	—	AAC7876
Aleiodes binkyi	Thailand	ASQSP864-08	JF962634	ADM6725
Aleiodes biscutus	Thailand	ASQSR117-11	JN278304	AAV7475
Aleiodes bobandersoni	Costa Rica	ASHYM2227-13	MH272220	AAA5372
Aleiodes bobwhartoni	Thailand	ASQSQ011-09	JF271188	AAH8788
Aleiodes borealis	USA	BBHYA2282-12	—	ACO1868
Aleiodes brunniguttatus	Thailand	ASQSP749-08	JQ388434	AAG7586
Aleiodes bucculentus	USA	BBHYG024-10	—	AAH8732
Aleiodes buoculus	USA	HYMBB606-09	HM423303	AAG8227
Aleiodes burrus	USA	ASQSP630-08	MH272375	ACF2634
Aleiodes buzurae	Thailand	ASQSQ484-09	HM435198	AAJ4035
Aleiodes buzuriduplus	Thailand	ASQSP1026-10	HQ551230	AET3833
Aleiodes buzuriquadruplus	Thailand	ASQSR192-11	JN278357	AAV7469
Aleiodes buzuritriplus	Thailand	ASQSR027-11	JN278254	AAV7506
Aleiodes cacuangoi	Ecuador	GBMIN74554-17	KX661405	AAM3865
Aleiodes calicis	Thailand	ASQSR095-11	JN278284	AAV7494
Aleiodes calvus	Thailand	ASQSR203-11	JN278367	AAV7467

Species	Country	BOLD	GenBank	BIN
Aleiodes cantherius	Sweden	GBMIN74555-17	KU682249	AEI9039
Aleiodes canus	Thailand	ASQSQ038-09	JQ388394	AAH8797
Aleiodes capillosus	Ecuador	ASQSQ732-10	HQ551416	AAM4968
Aleiodes caprinus	Thailand	ASQSP954-10	HQ551247	AAH8795
Aleiodes carbonarius	Hungary	ASQBR120-09	MK585853	AAE5121
Aleiodes carminatus	France	ASQBR151-09	KU682224	AAF1004
Aleiodes carolinagodoyae	Costa Rica	ASHYM317-08	JF792890	AAW1567
Aleiodes castaneus	Thailand	ASQSP215-08	JQ388461	ACF3848
Aleiodes caudalis	France	ASQSP1001-10	HQ551216	AEH9051
***Aleiodes ceres* sp.n.**	Argentina	GMARM1238-14	MH272298	ACN2401
***Aleiodes ceres* sp.n.**	Argentina	GMAGR178-15	OM611667	ACN2401
***Aleiodes ceres* sp.n. ES31A**	Brazil	—	OR235180	—
***Aleiodes ceres* sp.n. ES31T**	Brazil	—	OR235179	—
Aleiodes chamba	Thailand	ASQSP242-08	JF962579	AET3831
Aleiodes charlieobrieni	Costa Rica	ACGBA3908-13	MH272352	ACJ4200
Aleiodes chenduplus	Thailand	ASQSR270-11	JQ388447	AAV7456
Aleiodes cheni	Thailand	ASQSP423-08	JF962548	AAH8675
Aleiodes circumscriptus	UK	ASQBR174-09	MH272320	AAC0136
Aleiodes codon	Thailand	ASQBR373-09	JF962770	AAH8890
Aleiodes complexus	Thailand	ASQSP236-08	JQ388451	AAG7586
Aleiodes concoronarius	Thailand	ASQSP424-08	JQ388339	AAH8676
Aleiodes connudatum	Thailand	ASQSQ042-09	JQ388390	AAH8785
Aleiodes conpectenus	Thailand	ASQSR030-11	JQ388362	ACK0036
Aleiodes constriatum	Thailand	ASQSR181-11	JN278349	AAV7465
Aleiodes contemptus	Thailand	ASQSQ026-09	JQ388405	ADM6725
Aleiodes coriaceus	Sweden	ASQBR141-09	MK585885	AAC8307
Aleiodes coronopus	Thailand	ASQSQ010-09	JQ388416	AAH8787
Aleiodes corrusciput	Thailand	ASQSR119-11	JN278306	AEH9051
Aleiodes coxalis	UK	ASQSP553-08	MK585874	AAC1111
Aleiodes cramum	Thailand	ASQSR035-11	JN278259	AAH8638
Aleiodes crassipes	UK	ASQSP755-08	JF962844	ABX5850
Aleiodes cruentus	Germany	BCHYM7679-15	—	AAL7671
Aleiodes curticornis	Italy	GBAH20577-19	KU682236	AAE1750
Aleiodes damus	Thailand	ASQSP201-08	JQ388465	AAG7574
Aleiodes dangerlingi	Australia	HYAS008-10	HM914649	AAL8274

Species	Country	BOLD	GenBank	BIN
Aleiodes davefurthi	Costa Rica	ACGBA2891-12	MH272327	ACB2701
Aleiodes deathi	Thailand	ASQSP395-08	JQ388350	AAH8670
Aleiodes declanae	New Zealand	GBAHB479-14	KM106867	ACL9841
Aleiodes declanae	New Zealand	GBAHB456-14	KM106865	ACM0482
Aleiodes declanae	New Zealand	GBAHB423-14	KM106868	ACM0741
Aleiodes definus	Thailand	ASQSR053-11	JN278266	ACF3850
Aleiodes deyoyoi	Thailand	ASQSQ047-09	JQ388387	ADM6725
Aleiodes diarsianae	UK	ASQSP006-08	JF962600	ABZ6192
Aleiodes dimorphus	Thailand	ASQSQ049-09	JQ388385	AAG7584
Aleiodes dissector	Turkey	ASQBR115-09	JF957045	AAD4235
Aleiodes divergerus	Thailand	ASQSR125-11	JN278312	AAH8654
Aleiodes donwhiteheadi	Costa Rica	ASHYF1982-11	JQ575765	AAA5378
Aleiodes doylemckeyi	Costa Rica	PLCAK010-19	—	AAH8920
Aleiodes esenbeckii	Spain	GBMIN74556-17	KU682240	AAW1559
Aleiodes etvalinus	Thailand	ASQSR094-11	JN278283	ACF5418
Aleiodes faciei	Thailand	ASQSP196-08	JF963445	AAG7571
Aleiodes falloni	Ecuador	ASQSQ624-10	HQ551325	AAM5663
Aleiodes flannelfooti	Thailand	ASQSQ020-09	JQ388409	AAH8654
Aleiodes flavostriatus	Thailand	ASQSP849-08	JF962636	AAH8759
Aleiodes fortipes	France	ASQSP966-10	—	AAM0533
Aleiodes fovodeum	Thailand	ASQSR101-11	JN278290	AAV7473
Aleiodes frankhovorei	Costa Rica	ASHYE2251-11	JN278207	AAM5640
Aleiodes frosti	Ecuador	GBMIN74558-17	KX661403	AAM4918
Aleiodes fuscomedius	Thailand	ASQSP944-10	HQ551238	AAL9714
Aleiodes gaga	Thailand	ASQSR162-11	JN278336	ACF5419
Aleiodes gaspodei	Thailand	ASQSR174-11	JN278343	AAG7589
Aleiodes gasterator	France	ASQAS218-11	JF962846	ACF3778
Aleiodes gastritor	Germany	BCHYM7942-15	—	AAA7797
***Aleiodes gastritor* agg spG3**	UK	ASQSP101-08	MK585847	AEH9051
Aleiodes georgiae	Thailand	ASQSR041-11	JN278263	AAV7464
Aleiodes glabribasalis	Thailand	ASQSR184-11	JN278351	AAV7467
Aleiodes glandularis	South Africa	HYSAF058-12	MH234922	ABX3524
Aleiodes glutinum	Thailand	ASQSP231-08	JF963432	AAG7595
Aleiodes gonodontovorus	Costa Rica	ACGAZ1262-12	—	ACK7827
Aleiodes granulatus	Canada	JSHYP476-11	KR803687	AAH8781

Species	Country	BOLD	GenBank	BIN
Aleiodes grassator	UK	ASQSR276-11	—	ACE6137
Aleiodes griseimaculatus	Thailand	ASQSQ409-09	HM435161	AEG9999
Aleiodes guidaae	Thailand	ASQSP430-08	JQ388338	AAH8664
Aleiodes hei	Thailand	ASQSR208-11	JN278370	ACK0252
Aleiodes helenhippersonsae	Thailand	ASQSP953-10	HQ551246	AAH8969
Aleiodes henryhowdeni	Costa Rica	ACGAZ670-11	—	ABX5209
Aleiodes herrena	Thailand	ASQSR155-11	JN278329	AAG7586
Aleiodes hircus	Thailand	ASQSR180-11	JN278348	AAH8982
Aleiodes incisus	Thailand	ASQSR100-11	JN278289	AAV7470
Aleiodes inga	Costa Rica	ASHYE929-09	—	AAA5377
Aleiodes jakowlewi	Finland	ASQBR123-09	JF962849	AAW1571
Aleiodes jimwhitfieldi	Thailand	ASQSP500-08	JF271185	AAH8685
Aleiodes johnchemsaki	Costa Rica	ACGAZ566-11	MH272242	ABX5740
Aleiodes johnkingsolveri	Costa Rica	ASHYE2586-11	JQ574881	AAT8850
Aleiodes josesolanoi	Costa Rica	ACGBA10769-19	MW627567	AEB1913
Aleiodes juniorporrasi	Costa Rica	ACGBA10773-19	MW627570	AAV7490
Aleiodes kanyawarensis	Uganda	ASQBR351-09	EF115450	AAJ2477
Aleiodes kaydodgeae	Costa Rica	ACGBA10781-19	MW627545	AEB2985
Aleiodes klopfsteinae	Thailand	ASQSQ035-09	JQ388397	AAH8796
Aleiodes lavaeolous	Thailand	ASQSP220-08	JF963438	AAG7587
Aleiodes leptofemur	UK	ASQSP048-08	KU682230	AEI0523
Aleiodes lipwigduplicitus	Thailand	ASQSR027-11	JN278256	AAH8758
Aleiodes lipwigi	Thailand	ASQSR109-11	JN278298	ABZ2591
Aleiodes lobocarinus	Thailand	ASQSR089-11	JQ388358	AAV7492
Aleiodes luteosicarius	Ecuador	ASQSQ735-10	HQ551419	AAM4341
Aleiodes maculiput	Thailand	ASQSP387-08	JF962559	AAG7582
Aleiodes magratae	Thailand	ASQSR182-11	JN278350	AAV7466
Aleiodes malarius	Thailand	ASQSP442-08	JQ388334	AAH8681
Aleiodes malichi	Indonesia	INLEP3204-16	MH927903	ACE6023
Aleiodes manuelzumbadoi	Costa Rica	GMCRW566-13	MH272349	ACL0293
Aleiodes mayrabonillae	Costa Rica	ACGBA10662-19	—	AAD7873
Aleiodes mediofuscus	Thailand	ASQSR129-11	JN278315	AAL9721
Aleiodes mediomaculatus	Thailand	ASQSP415-08	JF962551	ABZ5311
Aleiodes megaophthalmos	Thailand	ASQSP948-10	HQ551241	AAL9715
Aleiodes melanopterus	Argentina	GMAGP017-15	OM597365	AAH8806

Species	Country	BOLD	GenBank	BIN
Aleiodes mericeti	Thailand	ASQSQ017-09	JQ388411	AAH8677
Aleiodes michelledsouzae	Costa Rica	ASHYH540-11	MH272180	ABA7287
Aleiodes microophthalmos	Thailand	ASQSP226-08	JF963435	AAG7591
Aleiodes mikeiviei	Costa Rica	MHMYC2414-15	MH272216	ACS9594
Aleiodes modestus	UK	ASQBR124-09	JF962850	AAJ2471
Aleiodes molecryptus	Thailand	ASQSP451-08	JF962540	ACI8591
Aleiodes morti	Thailand	ASQSQ561-09	HM435246	AAG7576
Aleiodes mubfsi	—	GBAH2900-07	EF115447	AAW1554
Aleiodes necsubson	Thailand	ASQSR090-11	JN278279	AAV7493
Aleiodes nigriceps	UK	ASQSP769-08	KU682243	AEI0523
Aleiodes nigricornis	Sweden	ASQBR185-09	KU682258	AEI0523
Aleiodes nivori	Thailand	ASQSP951-10	HQ551244	AAL9716
Aleiodes nobilis	Canada	CNRMD2133-12	KR783601	ABA6288
Aleiodes nobilis	UK	ASQSP375-08	MT606582	AAG7601
Aleiodes nonicones	Thailand	ASQSR118-11	JN278305	AAV7476
Aleiodes normwoodleyi	Costa Rica	ASHYB1459-09	MH272401	AAJ4076
Aleiodes nr. myoporhogas	Uganda	ASQSR297-11	MH272238	AAH8929
Aleiodes nunbergi	Austria	ASQBR930-18	MH272254	ADM0092
Aleiodes occimaculatus	Thailand	ASQSP210-08	JF963441	AAG7582
Aleiodes opus	Malaysia	ASQBR409-09	—	AAH8899
Aleiodes paenicarinus	Thailand	ASQSR084-11	JN278276	AAL9721
Aleiodes pallescens	Hungary	ASQSQ777-10	JN278215	AAV3392
Aleiodes pallidator	Canada	CNWLI343-12	KR789413	AAW1551
Aleiodes pallidicornis	Russia	ASQSP742-08	JF957043	AAH8742
Aleiodes pallimedius	Thailand	ASQSP952-10	HQ551245	AAL9717
Aleiodes palmae	Thailand	ASQSP213-08	JQ388462	AAG7575
Aleiodes palmatipes	Thailand	ASQSR076-11	JN278269	AAV7478
Aleiodes pammitchellae	Costa Rica	ACGBA8715-18	—	AAD7872
Aleiodes pappi	Kenya	BBTH766-17	MH234942	ADH4041
Aleiodes parabuzurae	Thailand	ASQSP1006-10	HQ551220	AAH8682
Aleiodes pauljohnsoni	Costa Rica	ASHYD4081-14	MH272372	ACM2562
Aleiodes paulmarshi	Taiwan	ASQSP293-08	—	ABY5567
Aleiodes pectopulicis	Thailand	ASQSP463-08	JF962535	AAH8677
Aleiodes pectunguis	Thailand	ASQSP389-08	JF962558	AAG7592
Aleiodes pectunguisella	Thailand	ASQSP228-08	JQ388453	AAG7592

Species	Country	BOLD	GenBank	BIN
Aleiodes penultimoluteus	Thailand	ASQSP427-08	JF962546	ACI8591
Aleiodes phantasmatis	Thailand	ASQSP195-08	JF963446	AAG7570
Aleiodes pictus	Austria	ASQSP543-08	KU682242	AEI0523
Aleiodes pinnulae	Thailand	ASQSP950-10	HQ551243	AAH8677
Aleiodes placidus	Thailand	ASQSP198-08	JQ388468	AAG7573
Aleiodes politiceps	USA	BBHYA1894-12	—	AAG8054
Aleiodes polititergus	Thailand	ASQBR369-09	JF962772	AAH8888
Aleiodes ponderi	Thailand	ASQSP956-10	HQ551248	AAL9718
Aleiodes postmaculus	Thailand	ASQSR130-11	JN278316	AAV7480
Aleiodes praetor	Bulgaria	ASQSQ728-10	MT606459	AAM0534
Aleiodes prillae	Thailand	ASQSP1008-10	HQ551221	AAH8784
Aleiodes probuzurae	Taiwan	ASQSP302-08	—	AAH8658
Aleiodes procarinatus	Thailand	ASQSP866-08	JF962632	AAH8761
Aleiodes procoronarius	Thailand	ASQSP448-08	JQ388330	AAU4869
Aleiodes pronopus	Thailand	ASQSP418-08	JQ388342	AAG7567
Aleiodes propodealis	Thailand	ASQSR104-11	JN278293	ACE2989
Aleiodes propodocarinus	Thailand	ASQSR083-11	JN278275	AAV7460
Aleiodes protocastaneus	Thailand	ASQSR151-11	JN278326	AAV7486
Aleiodes pseudoterminalis	Canada	ASQSP921-08	MH272291	AAG7629
Aleiodes pteppicymoni	Thailand	ASQSQ031-09	JQ388400	AAH8794
Aleiodes ptraci	Thailand	ASQSR131-11	JN278317	AAV7481
Aleiodes punctipes	UK	GBAH4091-09	EU979587	AAW1553
Aleiodes quadrum	France	ASQBR939-18	MH272232	ADM1859
Aleiodes rectanguliguttatus	Thailand	ASQSR126-11	JN278313	AAV7479
Aleiodes reticulisoma	Thailand	ASQSR124-11	JN278311	ABU5596
Aleiodes ridcullyi	Thailand	ASQSP865-08	JF962633	AAH8760
Aleiodes rincewindi	Thailand	ASQSP206-08	JF963442	AAG7579
Aleiodes rivulus	Thailand	ASQSR210-11	JN278372	AAV7472
Aleiodes roberti	Thailand	GBAHB1492-18	MH496739	ADM5100
Aleiodes rocioecheverri	Costa Rica	ASQSQ663-10	HQ551360	AAM5673
Aleiodes ronaldzunigai	Costa Rica	ACGBA10244-19	MW627568	AAH8707
Aleiodes rosewarnerae	Costa Rica	ASHYM276-08	—	AAW1573
Aleiodes ruficornis	Canada	CNMIH1590-14	KR411295	ACV3273
Aleiodes ruficornis	UK	ASQBR128-09	—	AAC2552
Aleiodes rufipes	Canada	CNKLC2424-14	KR785209	AAC8306

Species	Country	BOLD	GenBank	BIN
Aleiodes rufomedius	Thailand	ASQSP433-08	JF962544	AAH8678
Aleiodes rugoscutus	Thailand	ASQSQ501-09	HM435212	AAH8902
Aleiodes rugulosus	France	ASQSP011-08	—	AAF4165
Aleiodes ryrholmi	Sweden	ASQBR322-09	JF962792	AAH8874
Aleiodes sacharissa	Thailand	ASQSQ051-09	JQ388384	AEW0382
Aleiodes scottshawi	Thailand	ASQSQ063-09	JQ388378	AAH8800
Aleiodes selachiii	Thailand	ASQSQ048-09	JQ388386	AAH8798
Aleiodes seriatus	Honduras	GMHDE254-13	—	AAH8906
Aleiodes shakirae	Costa Rica	ACGBA10217-19	—	AEF4695
Aleiodes sibiricus	Sweden	ASQBR158-09	MH272159	AEH9265
Aleiodes similicodon	Thailand	ASQSQ411-09	HM435163	AAH8970
Aleiodes similis	Austria	ASQSQ727-10	HQ551413	AEI0523
Aleiodes smithi	Canada	OPPKI160-17	—	AAH8776
Aleiodes songsi	Thailand	ASQSP383-08	JF962560	AAH8666
Aleiodes sophieae	Thailand	ASQSQ362-09	—	AAH8683
Aleiodes sp signatus01	Sweden	ASQBR931-18	MH272383	AAA7796
Aleiodes spurivena	Vietnam	ASQSP145-08	JF962592	AAH8635
Aleiodes spurivenaduplus	Vietnam	ASQSP961-10	HQ551252	AAH8635
Aleiodes steveashei	Costa Rica	ACGBA1344-12	—	AAJ4092
Aleiodes stibbonsi	Thailand	ASQSR170-11	JN278340	AAV7462
Aleiodes stohelit	Thailand	ASQSP221-08	JF963437	AAG7588
Aleiodes subemarginatus	Thailand	ASQSQ357-09	JQ388371	AET3831
Aleiodes subfuscomedius	Thailand	ASQSR036-11	JN278260	AAL9721
Aleiodes subson	Thailand	ASQSP240-08	JQ388436	AAG7572
Aleiodes sutthisani	Thailand	ASQSR081-11	JN278273	AAG7599
Aleiodes terminalis	Canada	AGAKS1339-17	—	AAG5007
Aleiodes terryerwini	Costa Rica	ACGBA7877-17	MH496728	ADJ0647
Aleiodes testaceus	France	ASQSP116-08	KU682227	AAC2561
Aleiodes tetrarugulosus	Thailand	ASQSQ413-09	HM435165	AAH8971
Aleiodes texanus	USA	ASQBR512-09	JF962866	AAW1587
Aleiodes thirakupti	Thailand	ASQSR110-11	JN278299	AAH8957
Aleiodes tmaliaae	Thailand	ASQSQ416-09	HM435167	AES6605
Aleiodes tobiasi	Thailand	ASQSP194-08	JF963447	AAG7569
Aleiodes trevelyanae	Uganda	ASQBR350-09	—	AAF3781
Aleiodes trianguliscleroma	Malawi	BBTH765-17	MH234926	ADH6211

Species	Country	BOLD	GenBank	BIN
Aleiodes tricoloripes	Thailand	ASQSP381-08	JQ388353	AAH8665
Aleiodes turcicus	Turkey	ASQAS219-11	JF962613	ABY8565
Aleiodes turgidipalpus	Thailand	ASQSP224-08	JQ388456	AAG7590
Aleiodes ungularis	France	ASQSP757-08	JF903055	AAW1588
Aleiodes unipunctator	Canada	BBHYE396-10	HQ552347	AAE5902
Aleiodes valinus	Thailand	ASQSQ519-09	HM435229	AAG7598
Aleiodes variifemurus	Thailand	ASQSR054-11	JN278267	AAV7489
Aleiodes varius	Russia	ASQSP990-10	HQ551275	AAM0544
Aleiodes vetinarii	Thailand	ASQSP153-08	JF962590	AEU2159
Aleiodes vietuput	Thailand	ASQSR187-11	JN278353	AAV7468
Aleiodes willsflowersi	Costa Rica	ASHYC4711-10	HQ548643	AAM1704
Aleiodes wyomingensis	USA	HYMBC221-10	—	AAG8089
Aleiodes yalaensis	Thailand	ASQSR194-11	JQ388445	AAU1308
Aleiodes zuburae	Thailand	ASQSQ426-09	HM435172	AAH8973
Heterogamus dispar	UK	PEDIO346-09	MT639394	AAE3845
Heterogamus donstonei	Costa Rica	PLVAI691-20	—	AER5192
Heterogamus excavatus	Sweden	ASQBR935-18	MH272379	AAX0964
Heterogamus fasciatipennis	Finland	ASQSP523-08	—	AAE5420
